# ASO-mediated knock-down of GPNMB in mutant-*GRN* and in *Grn*-deficient peripheral myeloid cells disrupts lysosomal function and immune responses

**DOI:** 10.1186/s13024-025-00829-w

**Published:** 2025-04-08

**Authors:** Rebecca L. Wallings, Drew A. Gillett, Hannah A. Staley, Savanna Mahn, Julian Mark, Noelle Neighbarger, Holly Kordasiewicz, Warren D. Hirst, Malú Gámez Tansey

**Affiliations:** 1 Department of Neuroscience, Center for Translational Research in Neurodegenerative Disease, College of Medicine, University of Florida, McKnight Brain Institute, Gainesville, FL 32610 USA; 2 Department of Neurology, Fixel Institute for Neurological Diseases, University of Florida Health, Gainesville, FL 32608 USA; 3 Neurology, Ionis Pharmaceuticals, 2855 Gazelle Court, Carlsbad, CA 92010 USA; 4 Neurodegenerative Diseases Research Unit, Biogen, 115 Broadway, Cambridge, MA 02142 USA; 5 Current address: Department of Neurology, School of Medicine, Stark Neurosciences Research Institute, Indiana University, Indianapolis, IN USA; 6 Current address: DaCapo Brainscience, 700 Main Street, Cambridge, MA 02139 USA

**Keywords:** Antisense oligonucleotide, Progranulin, Frontotemporal dementia, Neurodegeneration, GPNMB, Immune response, Macrophages, Lysosome

## Abstract

**Background:**

GPNMB has been discussed as a potential therapeutic target in *GRN*-mediated neurodegeneration, based on the observed reproducible upregulation in FTD-*GRN* cerebrospinal fluid (CSF) and post-mortem brain. However, the functional impacts of up-regulated GPNMB are currently unknown, and it is currently unclear if targeting GPNMB will be protective or deleterious. Increases in GPNMB seen in FTD-*GRN* are reproduced in brains of aged *Grn*-deficient mice. Importantly, although brains of young *Grn*-deficient mice do not exhibit upregulated Gpnmb expression, peripheral immune cells of these mice exhibit increased Gpnmb expression as young as 5-to-6 months, suggesting the effects of *Grn*-deficiency in the periphery proceed those in the brain. *Grn*-deficiency is known to alter peripheral immune cell function, including impaired autophagy and altered cytokine secretion. GPNMB has potential effects on these processes, but has never been studied in peripheral immune cells of patients or preclinical models. Informing the functional significance of GPNMB upregulation in *Grn*-deficient states in myeloid cells has potential to inform GPNMB as a therapeutic candidate.

**Methods:**

The effects of GPNMB knock-down via antisense oligonucleotide (ASO) were assessed in peripheral blood mononuclear cells (PBMCs) from 25 neurologically healthy controls (NHCs) and age- and sex-matched FTD-*GRN* patients, as well as peritoneal macrophages (pMacs) from progranulin-deficient (*Grn -/-*) and B6 mice. Lysosomal function, antigen presentation and MHC-II processing and recycling were assessed, as well as cytokine release and transcription.

**Results:**

ASO-mediated knock-down of *GPNMB* increased lysosomal burden and IL1β cytokine secretion in FTD-*GRN* carriers and NHCs monocytes. ASO-mediated knock-down of *Gpnmb* in *Grn*-deficient macrophages decreased lysosomal pan-cathepsin activity and protein degradation. In addition, ASO-mediated knock-down of *Gpnmb* increased MHC-II surface expression, which was driven by decreased MHC-II uptake and recycling, in macrophages from *Grn*-deficient females. Finally, ASO-mediated knock-down of *Gpnmb* dysregulated IFN$\gamma$-stimulated IL6 cytokine transcription and secretion by mouse macrophages due to the absence of regulatory actions of the Gpnmb extracellular fragment (ECF).

**Conclusions:**

Our data herein reveal that GPNMB has a regulatory effect on multiple immune effector functions, including capping inflammation and immune responses in myeloid cells, potentially via secretion of its ECF. Therefore, in progranulin-deficient states, the marked upregulation in GPNMB transcript and protein may represent a compensatory mechanism to preserve lysosomal function in myeloid cells. These novel findings indicate that targeted depletion of GPNMB in FTD-*GRN* would not be a rational therapeutic strategy because it is likely to dysregulate important immune cell effector functions mediated by GPNMB. Specifically, our data indicate that therapeutic strategies inhibiting GPNMB levels and/or activity may worsen the effects of GRN deficiency.

**Supplementary Information:**

The online version contains supplementary material available at 10.1186/s13024-025-00829-w.

## Background

Progranulin (PGRN) is a secreted glycoprotein encoded by the *GRN* gene that is internalized via sortilin and prosaposin receptor-binding and taken to the lysosome where it is cleaved by intracellular cathepsins into its seven-and-a-half subunits called granulins [1–4]. Homozygous *GRN* mutations cause the lysosomal storage disorder (LSD) neuronal ceroid lipofuscinosis [5], whilst heterozygous mutations are associated with frontotemporal dementia (FTD-*GRN*) [6–8]. As well as FTD and LSDs, specific *GRN* variants have also been implicated in both Alzheimer’s Disease (AD) [9–11] and Parkinson’s Disease (PD) [12]. The effects of these pathogenic variants have been reported to dysregulate PGRN expression through nonsense-mediated decay, with a decrease in *GRN* mRNA and PGRN protein in patient samples across these diseases [13–16]. A loss of PGRN expression, and therefore function, is associated with increased risk for neurodegenerative diseases, and understanding mechanisms associated with *GRN* deficiency may aid in future therapeutic development.

Glycoprotein non-metastatic B (GPNMB) is a transmembrane type I protein that is highly expressed in both macrophages and microglia [17] and is one of the most upregulated transcripts and proteins in PGRN-deficient states [18]. Specifically, recent work has identified an increase in GPNMB in *GRN*-FTD patient brain lysate relative to both non-FTD controls and other FTD-related mutations [18]. Furthermore, an age-dependent increase in the expression of Gpnmb protein has been reported in *Grn -/-* mice, with brain lysates from 18-month old, but not 3-month old, *Grn -/-* mice exhibiting increased Gpnmb expression relative to B6 controls [18]. This suggests that Gpnmb upregulation in the CNS may be a component of later stages of disease progression. Importantly, our group demonstrated that Gpnmb is dysregulated in peripheral macrophages in the absence of PGRN at a significantly earlier time point (5-to-6 months) than previously reported in the CNS [19], highlighting that PGRN deficiency leads to early immune dysregulation in the periphery that pre-empts dysregulation in the CNS.

While it is currently unclear why GPNMB is elevated in human FTD-*GRN* patients and *Grn* -/- mice, GPNMB has been discussed as a potential therapeutic target in PGRN-mediated neurodegeneration [20]. Therefore, it is vital for the field to firmly establish whether targeting GPNMB will elicit beneficial or deleterious effects on immune system function and brain health. One intriguing possibility is that upregulation of GPNMB expression is driven by lysosomal dysfunction. This idea is supported by previous reports that lysosomal stress causes upregulation of GPNMB in macrophages [21]. GPNMB is also elevated in the *substantia nigra* of patients with PD [22], a neurodegenerative disease increasingly linked to lysosome dysfunction [23]. Moreover, chemical [22] or genetic inhibition [24] of β-glucocerebrosidase (GCase) activity, leading to perturbed lysosomal function, also causes increased expression of Gpnmb. Importantly, PGRN deficiency reduces GCase activity [25, 26], suggesting that the lysosomal dysfunction could be a proximal cause of GPNMB upregulation in *Grn* -/- mice and FTD-*GRN* patients.

It has also been suggested that GPNMB upregulation may occur in myeloid cells as a means to modulate or cap inflammation [17]. GPNMB has been reported to have an important anti-inflammatory role and is vital for response resolution. For example, RAW264.7 cells overexpressing GPNMB showed diminished levels of IL6 and IL12 cytokine release after IFNγ/LPS treatment when compared to controls [27], and primary human periodontal ligament cells (hPDLCs) overexpressing GPNMB showed decreased TNF and IL12 release after LPS treatment relative to controls [28]. Given the link between inflammation and PGRN deficiency [18, 20, 29, 30], it is possible, therefore, that GPNMB may be upregulated to help curb or dampen inflammation.

The aim of this study is to assess the effects of GPNMB knock-down via antisense oligonucleotides (ASOs) on both lysosomal function and immune cell responses in peripheral macrophages from 5-to-6-month-old *Grn* -/- mice and PBMCs from FTD-*GRN* patients to establish a functional proof of concept on whether GPNMB-targeting therapies would be deleterious or beneficial in the context of FTD-*GRN*. Importantly, we will specifically focus on peripheral myeloid cells given our recent findings demonstrating that upregulation of Gpnmb in *Grn* -/- mice is detectable much earlier in the peripheral immune system relative to the CNS [19], and prior to the onset of cognitive deficits in these mice which has been reported to occur at 13–to-16-months-of-age [19, 31]. This study will utilize an ASO-based method of knocking down GPNMB, as opposed to genetic ablation, because of reports that genetic ablation of *Gpnmb* does not alter synuclein-related pathology [32], which we posit may be due to an upregulation of compensatory genes in a *Gpnmb*-null environment. Furthermore, IFNγ will be used to stimulate myeloid cell to assess immune responses such as cytokine release and antigen presentation, which typically occur during an immune response to an inflammatory stimulus.

## Methods

### Human Peripheral Blood Mononuclear Cells (PBMCs)

Samples from the National Centralized Repository for Alzheimer’s Disease and Related Dementias (NCRAD) were used. Cryopreserved peripheral blood mononuclear cells (PBMCs) from *GRN*-FTD and age- and sex-matched neurologically healthy controls (*n* = 25 per participant group) were received and kept in liquid nitrogen cryostorage until use. See Tables 1 and 2, and Supplementary File 1 for participant demographics.

**Table 1:**
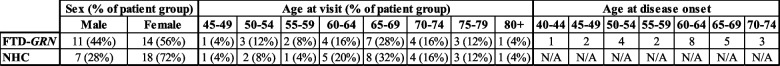
Patient demographics. NHC = neurologically heathy control, N/A = not applicable

**Table 2:**
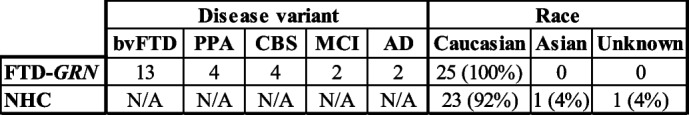
Patient demographics. NHC = neurologically heathy control, N/A = not applicable. bvFTD = Behavioral variant frontotemporal dementia, PPA = Primary progressive aphasia, CBS = Corticobasal syndrome, MCI = Mild Cognitive Impairment, AD = Alzheimer’s Disease

### Human PBMC cryorecovery

For cryorecovery, PBMCs were retrieved from liquid nitrogen, thawed at 37 °C, slowly added to 37 °C filter sterilized complete culture media (RPMI 1640 media, 10% heat-inactivated fetal bovine serum (FBS), 1 mM Penicillin–Streptomycin) and pelleted via centrifugation at 90 × *g* for 10 min at room temperature. The supernatant was removed and cells were resuspended in 37 °C MACS buffer (PBS, 0.5% bovine serum albumin, 20 mM EDTA, pH 7.2) cell counting using Trypan blue exclusion.

### Nucleofection and plating of human PBMCs

Cells were aliquoted into 50 mL falcon tubes with 1 × 10^6^ cells per nucleofection reaction. Cells were centrifuged at 90 × *g* for 10 min at 4 °C. The supernatant was carefully aspirated so as not to disturb the cell pellet, and cells were resuspended in nucleofection buffer (acclimated to room temperature; Lonza; P3 Primary Cell 4D-Nucleofector X Kit S; V4XP-3032) containing 1 µM *GPNMB-*targeting ASO or control ASO per 20 µL (ASOs provided by Ionis, sequences detailed in Table 3), to a final concentration of 1 × 10^6^ cells per 20 µL. 20 µL of cells were transferred to each Nucleocuvette, which was then placed into a 4D-Nucleofector® X Unit (Lonza) and pulsed using the EO 115 pulse code. After nucleofection, 400 µL of growth media (acclimated in incubator 1-h prior) was added to each Nucleocuvette and cells transferred to 24-well plates at a final concentration of 1 × 10^6^/mL, with 5 × 10^5^ cells per well. Cells were left to incubate for 24-h, after which cells were stimulated with vehicle or 100 U IFNγ (Peprotech) for 18-h.

**Table 3:**
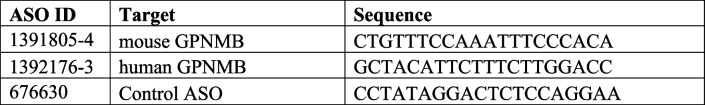
ASO sequences

### Flow cytometry of human PBMCs

After cryorecovery and nucleofection, PBMCs for flow cytometry were transferred to a v-bottom 96-well plate (Sigma, CLS3896-48EA) and centrifuged at 300 × *g* for 5 min at 4 °C. Cells were resuspended in 50μL growth media containing Lysotracker™ Red DND-99 and BMV109 (Vergent Bioscience), both at 1:1000 dilution, and incubated at 37 °C for 1-h. Cells were centrifuged for 5 min at 300 × *g* at 4 °C and washed in PBS × 2. Cells were then resuspended in 50 µL of PBS containing diluted fluorophore-conjugated antibodies (see Table 4) and incubated in the dark at 4 °C for 20 min. Cells were centrifuged at 300 × *g* for 5 min at 4 °C and washed in PBS × 2. Cells were fixed in 50 µL of 1% paraformaldehyde (PFA) at 4 °C in the dark for 30 min. Cells were centrifuged at 300 × *g* for 5 min and resuspended in 200 µL FACs buffer (PBS, 0.5 mM EDTA, 0.1% sodium azide). Cells were taken for flow cytometry on a MACS Quant Analyzer (Miltenyi). A minimum of 100,000 events were captured per sample and data were analyzed using FlowJo version 10.6.2 software (BD Biosciences). When validating flow cytometry panels and antibodies, fluorescence minus one controls (FMOCs) were used to set gates and isotype controls were used to ensure antibody-specific binding. Gating strategy is depicted in Sup. Figure 2A.

**Table 4:**
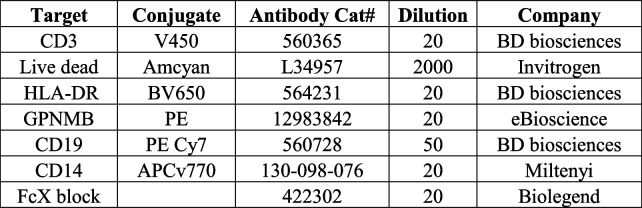
Human PBMC flow cytometry antibody panel

### Animals

*Grn* knock-out (*Grn* -/-) mouse strains have previously been characterized [30] and were maintained at 22 °C at 60–70% humidity and animals were kept in a 12-h light/dark cycle in the McKnight Brain Institute vivarium at the University of Florida. C57BL/6 littermate controls were used for all studies, with *Grn* -/- and C57BL/6 controls cohoused. All animal procedures were approved by the University of Florida Institutional Animal Care and Use Committee (IACUC #201,910,870) and were in accordance with the National Institute of Health Guide for the Care and Use of Laboratory Animals (NIH Publications No. 80–23) revised 1996. Male and female mice were aged to 5–6-months old and sacrificed via cervical dislocation or decapitation.

### Mouse peritoneal macrophages harvest and plating

Peritoneal macrophages (pMacs) were harvested from mice which had received a 1-mL intraperitoneal administration of 3% Brewer thioglycolate broth 72-h prior collection. Mice also received Buprenorphine Sustained-Release every 48-h for pain relief. Mice were sacrificed via cervical dislocation and abdomen sprayed with 70% ethanol. The skin of the abdomen was split along the midline, taking care to avoid puncturing or cutting the abdominal cavity. 10 mL of cold RPMI media (Gibco; 11,875,119) was injected into the peritoneal cavity using a 27G needle. After gentle massaging of the peritoneal cavity, as much fluid was withdrawn as possible from the peritoneal cavity using the 25G needle and a 10-mL syringe. Aspirated fluid was passed through a 70uM nylon filter onto 50 mL Falcon tubes and pre-wet with 5 mL of HBSS^−/−^. Filters were then washed twice with 5 mL of HBSS^−/−^ and then tubes centrifuged at 400 × *g* for 5 min at 4 °C. Supernatant was aspirated and pellet resuspended in 3 mL pre-warmed growth media (RPMI, 10% FBS, 1% Pen-Strep). Cells were counted and viability recorded using trypan-blue exclusion on an automated cell-counter (Countess™; Thermo). Volume growth media was adjusted so that cells were plated at 5 × 10^5^/mL in 6-, 24- or 96-well plates depending on the intended assay. Cells were incubated at 37 °C, 5% CO2 for a minimum of 3 h to allow macrophages to adhere to the plastic. Wells were washed twice with sterile PBS to remove non-adherent cells and new, pre-warmed growth media added. For cells requiring *ex-vivo* stimulation, 100U of IFNγ (R&D) or vehicle (H_2_O) was added for 18-h. Protocols available at https://dx.doi.org/10.17504/protocols.io.j8nlkoyoxv5r/v1.

### Mouse peritoneal macrophage nucleofection and plating

Peritoneal macrophages were harvested from mice as previously described. Once passed through 70uM nylon filter, tubes were centrifuged at 90 × *g* for 10 min at 4 °C. Cells were resuspended and counted as previously described. Cells were aliquoted into 50 mL falcons with 1 × 10^6^ cells per nucleofection reaction. Cells were centrifuged at 90 × *g* for 10 min at 4 °C. Supernatant was carefully aspirated so not to disturb the cell pellet, and cells resuspended in nucleofection buffer (acclimated to room temperature; Lonza; P2 Primary Cell 4D-Nucleofector X Kit L; V4XP-2024) containing 1 µM anti-*Gpnmb* ASO or control ASO per 100 µL (sequences detailed in Table 3), to a final concentration of 1 × 10^6^ cells per 100 µL. 100 µL of cells were transferred to each Nucleocuvette, which was then placed into a 4D-Nucleofector® X Unit (Lonza) and pulsed using the CM 138 pulse code. After nucleofection, 400 µL of growth media (acclimated in incubator 1-h prior) was added to each Nucleocuvette and cells transferred to plates pre-coated with poly-D-Lysine (Sigma). Cells were left to incubate for 24-h, after which media was aspirated, cells washed and treatment started as previously described.

### Multiplexed immunoassays of inflammatory analytes in peritoneal macrophage conditioned media

V-PLEX mouse pro-inflammatory panel 1 kit (MesoScaleDiscovery; K15048D) or V-PLEX human pro-inflammatory panel 1 kit (MSD; K15049D) was used to quantify cytokines in conditioned media from murine peritoneal macrophages (pMacs) and patient PBMCs, respectively. Media was diluted 1:1 with MSD kit diluent and incubated at room temperature in the provided MSD plate with capture antibodies for 2 h as per manufacturer’s instructions. Plates were then washed × 3 with PBS with 0.05% Tween-20 and detection antibodies conjugated with electrochemiluminescent labels were added and incubated at room temperature for another 2 h with shaking. After 3 × washes with PBS containing 0.05% Tween-20, MSD read buffer was diluted to 2 × and added, and the plates were loaded into the QuickPlex MSD instrument for quantification. Results were normalized to total live cell counts as measured via flow cytometry.

### Flow cytometry of mouse peritoneal macrophages

Media was aspirated from cells and cells washed 3 times in sterile PBS, harvested, and transferred to a v-bottom 96-well plate (Sigma, CLS3896-48EA) and centrifuged at 300 × *g* for 5 min at 4 °C. Cells were resuspended in 50 µL of PBS containing diluted fluorophore-conjugated antibodies (see Table 5) and incubated in the dark at 4 °C for 20 min. Cells were centrifuged at 300 × *g* for 5 min at 4 °C and washed in PBS × 2. Cells were fixed in 50 µL of 1% PFA at 4 °C in the dark for 30 min. Cells were centrifuged at 300 × *g* for 5 min. For intracellular Gpnmb staining, cells were resuspended in 100 µL of permeabilization buffer (eBiosciences, 00–8333-56) and incubated on ice for 15 min. Conjugated anti-Gpnmb antibody (see Table 5) was added to each well and incubated on ice for 20 min. Cells were centrifuged at 300 × *g* for 5 min at 4 °C and washed in PBS × 2 before being resuspended in 200 µL FACs buffer (PBS, 0.5 mM EDTA, 0.1% sodium azide). Cells were taken for flow cytometry on a Macs Quant Analyzer (Miltenyi) or BD LSR Fortessa™ Cell Analyzer. A minimum of 100,000 events were captured per sample and data were analyzed using FlowJo version 10.6.2 software (BD Biosciences). When validating flow cytometry panels and antibodies, fluorescence minus one controls (FMOCs) were used to set gates and isotype controls were used to ensure antibody-specific binding. experiments. Protocol is available at https://dx.doi.org/10.17504/protocols.io.rm7vzx9x4gx1/v1.

**Table 5:**
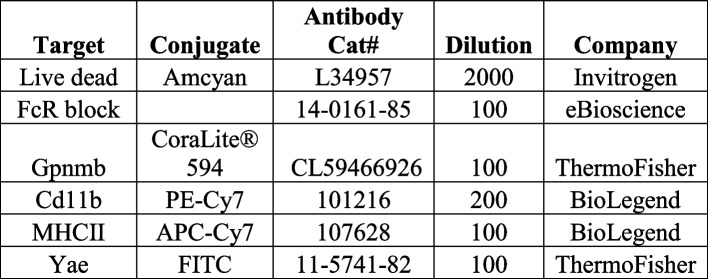
Mouse pMac flow cytometry antibody panel

#### DQ-BSA and BMV109 fluorescence microscopy

BMV109 Pan Cathepsin probe (Vergent Bioscience) and DQ red BSA (Invitrogen) were added to each well at a final concentration of 1 µG and 10 µG/mL, respectively, and cells were incubated at 37 °C for 1-h. Cells were washed 3 × DPBS^+/+^ and fixed for 10 min at room temperature in Invitrogen™ eBioscience™ Intracellular Fixation buffer. Cells were washed 3 × DPBS^+/+^ and incubated in 1 μg/ml DAPI (Life Technologies) for 10 min at room temperature in DPBS^+/+^. Cells were imaged using an EVOS™ M7000 (Invitrogen) at 20 × magnification. Image analysis was performed using Cellprofiler 4.2.5. Protocol available at https://dx.doi.org/10.17504/protocols.io.261gedk5ov47/v1.

#### ***MHC-II Ea***_***(52–68)***_*** peptide loading assay***

MHC II Ea chain (Ea) (52–68) peptide (Anaspec) was reconstituted in sterile distilled H_2_O to a final concentrate of 1 mg/mL. Once peritoneal macrophages had adhered to plates, 5 µg per well was added in growth media. Cells were incubated for 18-h and taken forward for flow cytometry. Protocol available at https://dx.doi.org/10.17504/protocols.io.14egn3r3pl5d/v1.

#### Immunoblotting

Media was aspirated and cells washed in PBS and lysed in RIPA buffer (50 mM Tris pH 8, 150 mM NaCl, 1% NP-40, 0.5% Na Deoxycholate, 0.1% SDS). Cell lysates were then centrifuged at 10,000 × *g* for 10 min at 4 °C. 6X Laemmli sample buffer added (Thermo Fisher) and samples were reduced and denatured at 95 °C for 5 min. Samples were loaded into 4–20% Criterion Tris–HCl polyacrylamide gels (BioRad) alongside Precision Plus Protein Dual-Color Ladder (Biorad) to determine target protein molecular weight. Electrophoresis was performed at 100 V for ~ 60 min and proteins transferred to a polyvinylidene difluoride (PVDF) membrane using a Trans-Blot Turbo Transfer System (BioRad) which utilizes Trans-Blot Turbo Midi PVDF transfer packs (BioRad) in accordance with manufacturer’s instructions. Prior to blocking, total protein was measured using Revert total protein stain (Licor) and imaged on the Odyssey FC imaging system (Licor). Membranes were then blocked in 5% non-fat milk in TBS/0.1% Tween-20 (TBS-T) for 1 h at room temperate and subsequently incubated with primary antibody (see Table 6) in blocking solution overnight at 4 °C. Membranes were washed with TBS-T (3 × 5 min) and incubated in horseradish peroxidase (HRP)-conjugated secondary antibody (1:1000) (BioRad) in blocking solution for 1 h. Membranes were washed in TBS-T (3 × 5 min) and developed using Super signal west Femto/Pico (Thermo). Membranes were imaged using the Odyssey FC imaging system and quantified using Image Studio Lite Version 5.2 (Licor).

**Table 6:**
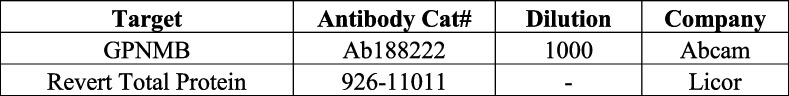
Western blotting antibodies

#### MHC-II antibody uptake assay

MHC-II uptake was adapted from [33]. Briefly, 100U IFNγ was added to cultured pMacs in 24-well plates and cells incubated in primary anti-MHC-II monoclonal antibody (mAB; BD Biosciences, see Table 7) at a final concentration of 5 μg/mL for 20 min at 4 °C in FACS buffer containing 10% normal goat serum, CD16/CD32 (Mouse BD Fc Block; BD Biosciences) at a final concentration of 1:100 and live/dead stain (see Table 7). Antibody solution was aspirated and cells were washed in ice-cold FACS buffer × 2. pMacs were incubated in prewarmed growth media at 37 °C for 0, 1 or 2 h to allow for the uptake of antibody-bound MHC-II complexes. “No endocytosis” controls were incubated in ice-cold FACS buffer and maintained on ice for 2 h. At each time point, internalization was stopped by adding 1 mL of ice-cold FACS buffer. Antibody-bound MHC-II that was not internalized was labelled with a fluorescent secondary antibody (see Table 7) at a final concentration of 5 μg/mL for 20 min on ice. Secondary antibody solution was aspirated and cells washed in ice-cold FACS buffer × 2. Cells were harvested and transferred to a v-bottom 96-well plate (Sigma, CLS3896-48EA) and centrifuged at 300 × *g* for 5 min at 4 °C. Cells were fixed in 50 µL of 1% PFA at 4 °C in the dark for 30 min. Cells were centrifuged at 300 × *g* for 5 min and resuspended in 200 µL FACs buffer. Cells were taken for flow cytometry on a Macs Quant Analyzer (Miltenyi) or BD LSR Fortessa™ Cell Analyzer. A minimum of 100,000 events were captured per sample and data were analyzed using FlowJo version 10.6.2 software (BD Biosciences).

**Table 7:**
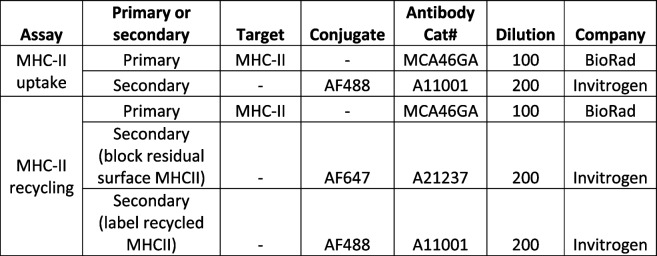
Antibodies for MHCII uptake and recycling assays

#### MHC-II recycling assay

MHC-II recycling was adapted from [33]. Briefly, 100 U IFNγ was added to cultured pMacs in 24-well plates, and cells incubated in primary anti-MHC-II monoclonal antibody (mAB; BD Biosciences, see Table 7) at a final concentration of 5 μg/mL for 1 h at 37 °C in FACS buffer to allow for the antibody-binding and subsequent uptake of MHC-II complexes. Antibody solution was aspirated and 1 mL of ice-cold FACS buffer added to each well to stop primary antibody uptake, and cells washed 2 × in ice-cold FACS buffer. To block any antibody-labelled MHC-II complexes that had not been internalized and was left on the plasma membrane, cells were incubated for 20 min on ice in FACS buffer containing goat anti-mouse IgG-Alexa 647 (see Table 7) at a final concentration of 5 μg/mL. Cells were washed 2 × with ice-cold FACS buffer and complete growth media added to each well. Cells were returned to 37 °C incubator for an additional 1-, 3- or 18-h. After each time point, 1 ml of ice-cold FACS buffer was immediately added to each well to stop recycling of MHC-II and cells washed × 2 with ice-cold FACS buffer. Cells were incubated for 20 min on ice in FACS buffer containing goat anti-mouse IgG-Alexa 488 (see Table 7) at a final concentration of 5 μg/mL. This step will label any primary antibody-labelled MHC-II, that had been taken up in the first incubation step, evading labelling with goat anti-mouse IgG-Alexa 647 in the blocking step and had been recycled back to the plasma membrane. Cells were harvested and transferred to a v-bottom 96-well plate (Sigma, CLS3896-48EA) and centrifuged at 300 × *g* for 5 min at 4 °C. Cells were fixed in 50 µL of 1% PFA at 4 °C in the dark for 30 min. Cells were centrifuged at 300 × *g* for 5 min and resuspended in 200 µL FACs buffer. Cells were taken for flow cytometry on a Macs Quant Analyzer (Miltenyi) or BD LSR Fortessa™ Cell Analyzer. A minimum of 100,000 events were captured per sample and data were analyzed using FlowJo version 10.6.2 software (BD Biosciences).

#### *Quantification of Gpnmb extracellular fragment *via* ELISA*

Conditioned media was collected from the pMacs at the indicated times. After being transferred to a 1.5 mL tube, the media was centrifugated at 10,000 × *g* for 10 min at 4 °C to remove any cell debris. The supernatant was transferred to a new tube and stored at -20 °C for later analysis. Endogenous Gpnmb ECF was measured using a Biotechne Gpnmb ELISA Kit (Biotechne/R&D Systems, DY2330) following manufacturer instructions with minor modifications. A four-parameter logistic (4-PL) curve was generated from the Gpnmb ECF standards included in the kit. The standard curve was created with an online 4-PL curve calculator from AAT Bioquest (https://www.aatbio.com/tools/four-parameter-logistic-4pl-curve-regression-online-calculator). The equation from the 4-PL curve was used to determine the concentration of Gpnmb ECF in the test samples.

#### RNA extraction and qPCR of inflammatory genes and Gpnmb

RNA was isolated using a Qiagen Mini-kit (Qiagen, category number 74104), following manufacturer instructions with minimal alterations. The concentration of BME was increased to a final concentration of 20uM to inhibit endogenous RNase activity. After elution, RNA concentration was determined using a Denovix spectrophotometer. cDNA was generated using a High-Capacity cDNA Reverse Transcription Kit (Applied Biosystems™, catalog number 4374966) following kit instructions. 2 × Universal SYBR Green Fast qPCR Mix (Abclonal, RK21203) was used with the indicated primers (see Table 8) for detection. A final mass of 6.25 ng of cDNA was used in each well. Each sample was run in triplicate per gene. Variance above 0.4 standard deviations from the sample’s average were excluded.

**Table 8:**
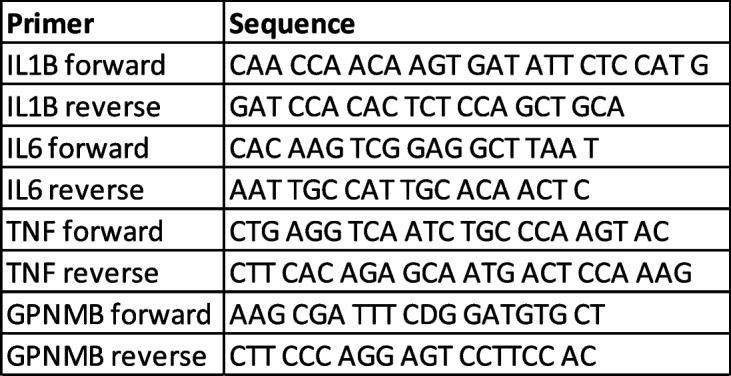
Primer sequences for RTqPCR

#### Statistics and data analysis

Statistics and data analyses were performed using IBM SPSS statistics 27 or GraphPad Prism 9. When assessing differences between ASO treatment groups when optimizing ASO conditions (in both human PBMCs and murine pMacs), one-way ANOVAs were performed. For flow cytometry, cytokine assessment and microscopy image analysis in pMacs where differences were assessed in 2 independent variables (mouse genotype and ex vivo treatment), two-way ANOVAs were performed. For flow cytometry and cytokine assessment in human PBMCs where differences were assessed in 2 independent variables (disease status and ex vivo treatment), two-way ANOVAs were performed. For flow cytometry-based pulse-chase assays in pMacs where differences were assessed in 3 independent variables (mouse genotype, ex vivo treatment and time), three-way ANOVAs were performed. The normal distribution of the data in this study was determined using the Shapiro–Wilk test. Homogeneity of Variance of the data in this study was determined using the Levene’s test. In instances when data did not fit parametric assumptions (heterogeneity of variance and/or non-normally distributed data), Kruskal–Wallis rank sum test was used, with Dunn’s test used for post-hoc analysis with Bonferroni correction. Post-hoc tests following ANOVAs were conducted using Bonferroni correction. Two-tailed levels of significance were used and p < 0.05 was considered statistically significant. * = *p* < 0.05, ** = *p* < 0.01, *** = *p* < 0.005, **** = *p* < 0.001. Graphs are depicted by means ± standard error of the mean (SEM). When deciding if data points are true statistical outliers versus a reflection of biological variability, we assume that data points that are ± 2 standard deviations of the mean of a data set are statistical outliers and are therefore excluded from data sets.

## Results

### Optimization of *GPNMB* knock-down via antisense oligonucleotides (ASOs) in human peripheral blood mononuclear cells (PBMCs)

Before assessing the effects of GPNMB knock-down on PBMC immune cell function and lysosomal homeostasis in FTD-*GRN* and control groups, the *GPNMB*-targeting ASO (Ionis) was first optimized in healthy control samples. PBMCs from neurologically healthy controls (NHCs) were nucleofected with 1, 2 or 5 μg of control or *GPNMB-*targeting ASO per reaction (1 × 10^6^ cells per reaction) and assessed for GPNMB expression levels. A significant reduction in GPNMB median fluorescence intensity (MFI) was observed in all cell types nucleofected with 1 μg *GPNMB*-targeting ASO relative to control ASO (Sup. Figure 1A-D). No significant effect of *GPNMB* ASO was observed at either of the higher concentrations, with these higher concentrations decreasing GPNMB even in control ASO conditions. A concentration of 1 μg was concluded as efficient to reduce GPNMB in human PBMC’s relative to control ASO. To ensure that nucleofection did not have adverse effects on inflammatory responses or lysosomal function that may confound the interpretation of result, PBMCs from NHCs were nucleofected with 1 μg control ASO per reaction (1 × 10^6^ cells per reaction) or left non-nucleofected to assess effects of nucleofection. Cells were then plated in the presence or absence of 100 U IFNγ for 18-h and inflammatory responses and lysosomal function assessed via flow cytometry. Nucleofection had no significant effect on live cell percentages, GPNMB expression, lysosomal function nor stimulation-dependent HLA-DR expression (Sup. Figure 1E-H). As no effects of nucleofection were observed, all experiments discussed here on directly compare the effects of *GPNMB* knock-down via ASO relative to control ASO, with no non-nucleofected control cells.

### FTD-*GRN* patient monocytes exhibit increased GPNMB expression and decreased stimulation-dependent HLA-DR expression and IL1β-secretion

PBMCs were collected from 25 FTD-*GRN* patients and 25 age- and sex-matched NHCs (See Tables 1 and 2, and Sup. File 1 for participant demographics) and sourced from the National Centralized Repository for Alzheimer’s Disease and Related Dementias (NCRAD). PBMCs were cryorecovered, nucleofected with 1 μg of *GPNMB* or control ASO (1 × 10^6^ cells per reaction), plated and allowed to recover for 24 h, after which 100 U IFNγ or vehicle control was added to each well and cells were incubated for 18-h (Sup. Figure 1I).

**Fig. 1:**
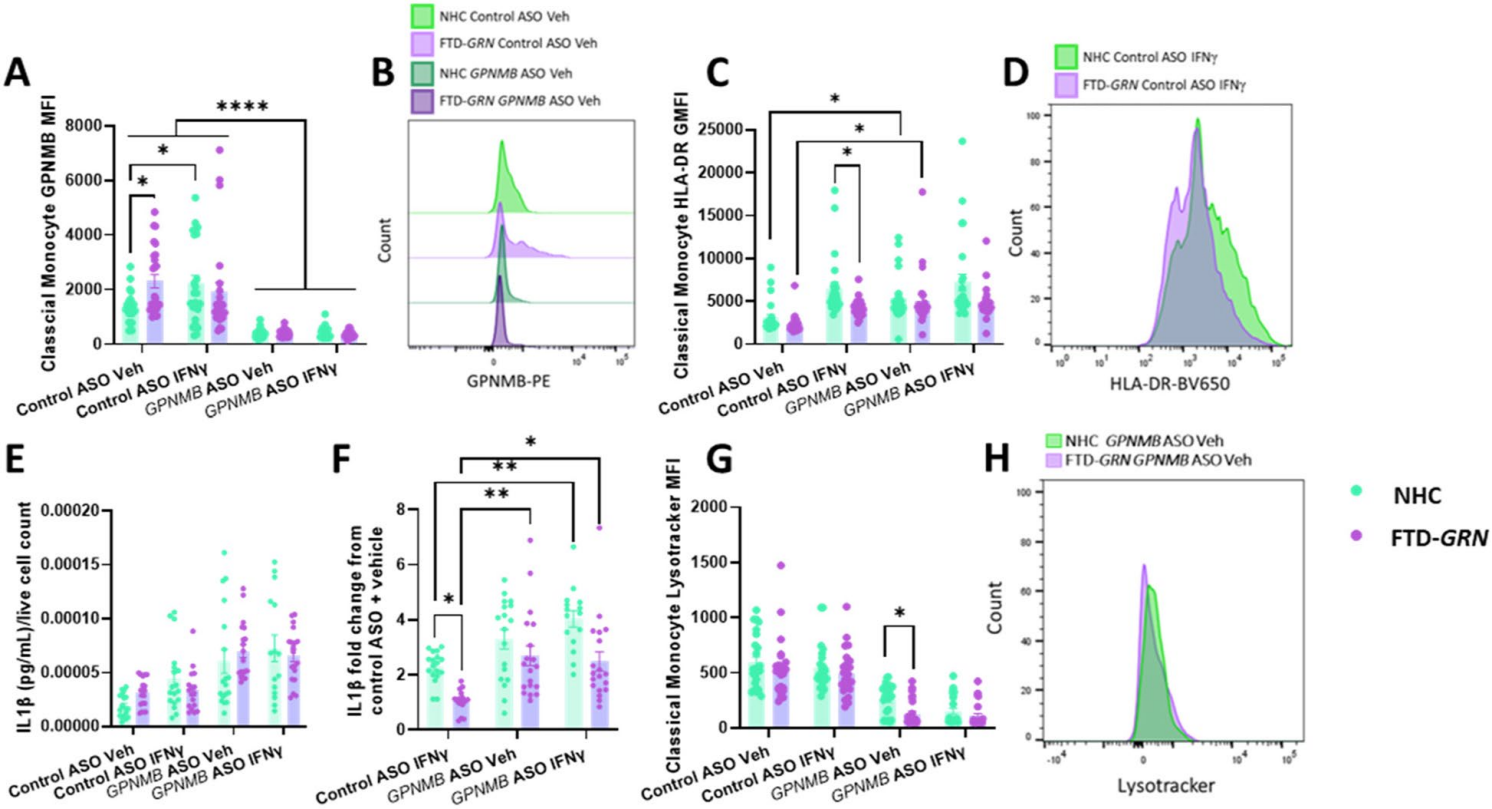
ASO-mediated knock-down of GPNMB increases HLA-DR expression and IL1β secretion in human PBMCs and imapirs lysosomal acidity in FTD-*GRN* monocytes. PBMCs from NHCs and FTD-*GRN* patients were nucleofected with control or *GPNMB*-targeting ASO, plated and allowed to rest for 24 h, followed by 18-h incubation in presence or absence of 100 U IFNγ and cells assessed via flow cytometry and media taken for cytokine quantification. GPNMB MFI was quantified in classical monocytes (**A**, **B**). HLA-DR was quantified in classical monocytes (**C**, **D**). IL1β release was quantified in media, normalized to live cell count (**E**) and fold-change from control ASO vehicle conditions calculated (**F**). Lysotracker MFI was quantified in classical monocytes (**G**, **H**). Bars represent mean ± SEM (N = 20–25 participants per disease state). Two-way ANOVA, Bonferroni post-hoc, * = *p* < 0.05, ** = *p* < 0.01, *** = *p* < 0.005, **** = *p* < 0.001

We first assessed GPNMB MFI in different PBMC immune cell subsets (Sup. Figure 2A) to assess effects of *GRN* mutations on GPNMB expression. It was observed that classical monocytes from FTD-*GRN* patients exhibited significantly increased GPNMB MFI in control ASO and vehicle conditions relative to NHCs (Fig. 1A, B). Regarding other immune cell subtypes, GPNMB MFI was also significantly increased in B cells from FTD-*GRN* patients relative to NHCs (Sup. Figure 2B), however no significant effects of patient group were seen in T cells (Sup. Figure 2C).

Given that many myeloid cell effector functions, such as antigen presentation and cytokine release, have been reported to be altered by *GRN* mutations and *Grn*-deficiency, we next sought to assess the effects of *GRN* mutations on effector functions of patient monocytes. Surprisingly, when quantifying HLA-DR extracellular expression in classical monocytes, a significant decrease in stimulation-dependent HLA-DR expression was observed in FTD-GRN classical monocytes relative to NHCs in control ASO conditions (Fig. 1C, D).

To determine if alterations in HLA-DR expression were accompanied by alterations in cytokine release, we performed multiplexed immunoassays on conditioned media from plated PBMCs stimulated with vehicle or IFNγ for 18-h after nucleofection. When measuring cytokine secretion from PBMCs normalized to the percentage of live cells, taken from flow cytometry data previously described, no significant differences were detected between FTD-*GRN* patients and NHCs (Fig. 1E, Sup. Figure 2D-G). However, when these values were quantified as a stimulation-dependent fold-change from baseline (control ASO vehicle conditions), secretion of IL1β specifically was decreased in the FTD-*GRN* relative to NHCs in control ASO conditions (Fig. 1F). Similar patterns were observed with TNF (Sup. Figure 2H) but no other cytokines (Sup. Figure 2I-K). Collectively, these data suggest that, in control ASO conditions, FTD-*GRN* monocytes exhibit increased GPNMB expression that is accompanied by blunted responses to IFNγ as indicated by decreased HLA-DR expression and IL1β secretion.

To assess the effects of *GRN* mutations, MFI of Lysotracker DND-99, which will accumulate in and stain sufficiently acidic lysosomes within cells, was quantified in classical monocytes. Interestingly, no significant differences in Lysotracker MFI were exhibited between patient groups in control ASO conditions (Fig. 1G).

### *GPMNB* knockdown in both FTD-*GRN* and NHC monocytes increases HLA-DR expression and IL1β secretion following IFNγ stimulation

GPNMB expression was quantified to assess the efficacy of ASO-mediated *GPNMB* knock-down in FTD-*GRN* patient PBMCs and those from NHCs. Indeed, *GPNMB* knock-down decreased GPNMB MFI levels in both vehicle and IFNγ treatments and patient groups in all cell types assessed (Fig. 1A, B; Sup. Figure 2B, C).

Interestingly, with ASO-mediated knock-down of GPNMB, no stimulation-dependent increase in HLA-DR was observed in classical monocytes from either FTD-*GRN* patients or NHCs due to a significant increase in HLA-DR expression in the vehicle *GPNMB* ASO conditions relative to control ASO. It appears therefore, that an increase in GPNMB levels is associated with suppressed stimulation-dependent changes in HLA-DR expression, and that knock-down of GPNMB increases HLA-DR expression in classical monocytes. Similarly, upon knock-down of GPNMB, IL1β fold-change from control ASO vehicle conditions was significantly increased in both patient groups, suggesting that GPNMB may exert anti-inflammatory effects, and knock-down of GPNMB increases the pro-inflammatory phenotype of PBMCs. No significant effects of GPNMB knock-down were observed for any other cytokine (Sup. Figure 2H-K).

### GPNMB knockdown reveals decrease in lysosomal acidity in FTD-*GRN* patient monocytes

Regarding lysotracker MFI, despite no significant differences being observed in control ASO conditions between FTD-*GRN* patient monocytes and those from NHCs, a significant decrease in Lysotracker MFI was observed in FTD-*GRN* classical monocytes relative to NHCs upon the knock down of *GPNMB* via ASOs in vehicle conditions (Fig. 1G, H). Such data suggest that an upregulation of GPNMB may be a compensatory mechanism to protect lysosomal function in FTD-*GRN* monocytes, with the subsequent loss of GPNMB predisposing patient cells to a disruption in proper lysosomal acidity.

### Optimization of *Gpnmb* knock-down via antisense oligonucleotides (ASOs) in primary murine peritoneal macrophages

To continue to investigate the effects of *Gpnmb* knock-down in myeloid cells in the context of PGRN deficiency, assays were repeated in pMacs from *Grn* -/- mice and B6 controls. *Gpnmb*-targeting and control ASOs were provided by Ionis. To optimize the use of these ASOs in pMacs, pMacs from 2-to-3-month-old B6 male mice were nucleofected with 2 different concentrations of *Gpnmb* or control ASO (2 × 10^6^ cells per reaction) and Gpnmb protein levels were assessed alongside non-nucleofected controls. A significant reduction in Gpnmb protein levels were observed in pMacs nucleofected with both concentrations of *Gpnmb*-targeting ASO, with no significant effect of control ASOs relative to non-nucleofected controls (Sup. Figure 3A, B). Moving forward, therefore, all experiments hereon were performed using the lower dose, at 1 µg, of control or *Gpnmb-*targeting ASO.

To ensure that nucleofection with this concentration of control ASO did not have adverse effects on inflammatory responses that may confound the interpretation of results, pMacs from 5-to-6-month-old, male and female, *Grn* -/- mice and B6 controls were nucleofected with control ASO or left non-nucleofected. Surface MHC-II expression at baseline and in response to IFNγ was quantified via flow cytometry. It is important to note that pMacs are not a homogenous population of cells, but rather a mix of small and large pMacs that can be distinguished by cd11b expression (SPMs and LPMs, respectively; Sup. Figure 3C). LPMs are resident to the peritoneal cavity and are traditionally thought of as phagocytic and responsible for the presentation of exogenous antigens, whilst SPMs are generated from bone-marrow-derived myeloid precursors which migrate to the peritoneal cavity and present a pro-inflammatory functional profile [34]. No significant differences were observed between non-nucleofected cells and those nucleofected with control ASO (Sup. Figure 3D-G). BMV109, a pan-cathepsin fluorescent probe, was used to quantify cathepsin activity in LPMs after nucleofection with a control ASO alongside non-nucleofected controls to assess effects of nucleofection on lysosomal function. No significant differences were observed between non-nucleofected and control ASO conditions in either genotypes or sexes (Sup. Figure 3H, I). Collectively, these data suggest that nucleofection with a control ASO at 1 µg does not significantly modify immune responses or lysosomal function in macrophages relative to non-nucleofected cells. Therefore, all experiments discussed herein.

directly compare the effects of Gpnmb knock-down via ASO relative to control ASO, with non-nucleofected control cells.

### *Grn*-deficient mouse macrophages display upregulation of Gpnmb and are responsive to inflammatory insults and ASO-mediated knock-down

**Fig. 2:**
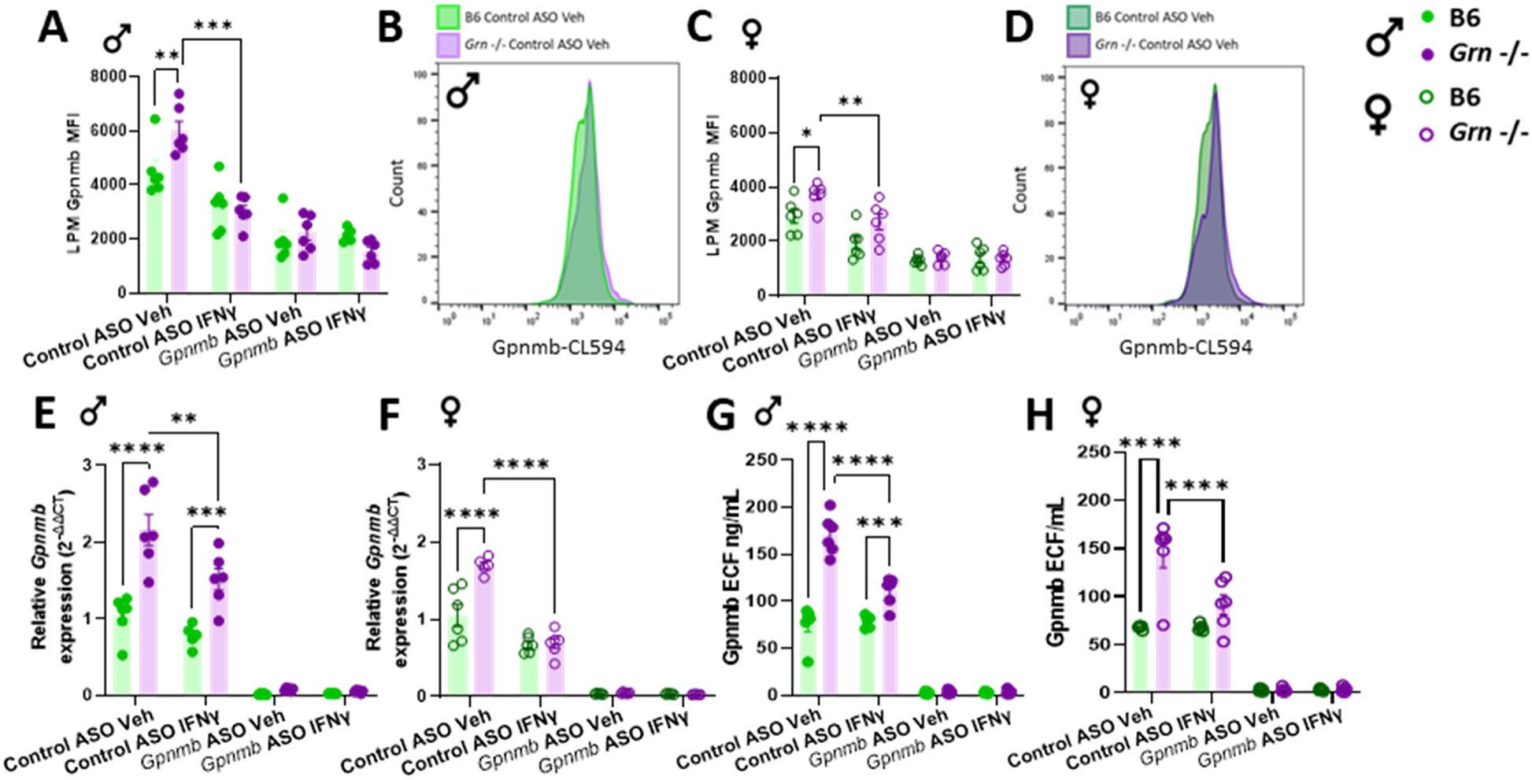
*Grn*-deficient mouse macrophages display upregulation of Gpnmb protein, transcript, and extracellular fragment (ECF) all of which are modulated by inflammatory insults and ASO-mediated knock-down. pMacs from B6 and *Grn* -/-, 5-to-6-month-old, male and female mice were nucleofected with control or *Gpnmb*-targeting ASO, plated and allowed to rest for 24 h, followed by 18-h incubation in presence or absence of 100 U IFNγ and cells assessed via flow cytometry or RNA extracted. Gpnmb MFI was quantified in LPMs from male and female mice (**A-D**). Total *Gpnmb* transcript levels were quantified in pMacs from male and female mice (**E**, **F**). Gpnmb ECF was quantified in media from pMacs from male and female mice (**G**, **H**). Bars represent mean ± SEM (N = 5–6 mice per genotype). Two-way ANOVA, Bonferroni post-hoc, * = *p* < 0.05, ** = *p* < 0.01, *** = *p* < 0.005, **** = *p* < 0.001

Gpnmb expression was first assessed in pMacs from 5-to-6-month-old *Grn* -/- mice and B6 controls nucleofected with control or *Gpnmb*-targeting ASO (Sup. Figure 3J). Both male and female mice were used and analyzed separately given the known sex-differences within the immune system [35]. Nucleofected cells were plated and incubated with 100U IFNγ for 18-h or a vehicle in order to assess effects of IFNγ on Gpnmb expression. A significant increase in Gpnmb expression was observed in both LPMs and SPMs from male and female *Grn* -/- mice relative to B6 controls in control ASO vehicle conditions (Fig. 2A-D; Sup Fig. 3K, L). Interestingly, IFNγ significantly reduced Gpnmb expression in LPMs and SPMs from male and female *Grn* -/- from vehicle treated conditions. ASO-mediated knock-down of *Gpnmb* reduced Gpnmb expression in LPMs and SPMs in both sexes, genotypes and treatments. Orthogonal qPCRs were carried out to confirm our findings, and mRNA transcript levels of total pMacs reflected what was observed via flow cytometry, with the exception that, in control ASO pMacs treated with IFNγ, male *Grn* -/- mice retained an increase in *Gpnmb* expression relative to B6 controls, although it was observed to be significantly decreased from vehicle controls (Fig. 2E, F). Collectively, this data demonstrate that Gpnmb is increased in LPMs and SPMs from 5-to-6-month-old, male and female, *Grn* -/- mice relative to B6 controls, and that Gpnmb can be downregulated upon pro-inflammatory stimulus or ASO-mediated knock-down of Gpnmb.

Gpnmb, as a membrane protein, can be cleaved and the extracellular fragment (ECF) acts as a paracrine factor to affect neighboring cells for modulating tumorigenesis and immunosuppression [36]. When Gpnmb ECF was quantified via ELISA in cell culture media, a significant reduction in Gpnmb ECF upon *Gpnmb*-targeting ASO nucleofection was observed in both genotypes and sexes relative to control ASO conditions (Fig. 2G, H). Interestingly, in control ASO conditions, Gpnmb ECF levels reflected cellular protein expression that were reported via flow cytometry, with significant increases observed in *Grn* -/- pMacs relative to B6 controls. Furthermore, Gpnmb ECF is significantly reduced in control ASO nucleofected *Grn* -/- pMacs in the presence of IFNγ relative to vehicle controls but still remained significantly elevated relative to B6 controls.

### ASO-mediated knock-down of *Gpnmb* decreases lysosomal health and protein degradation in *Grn*-deficient female macrophages

**Fig. 3:**
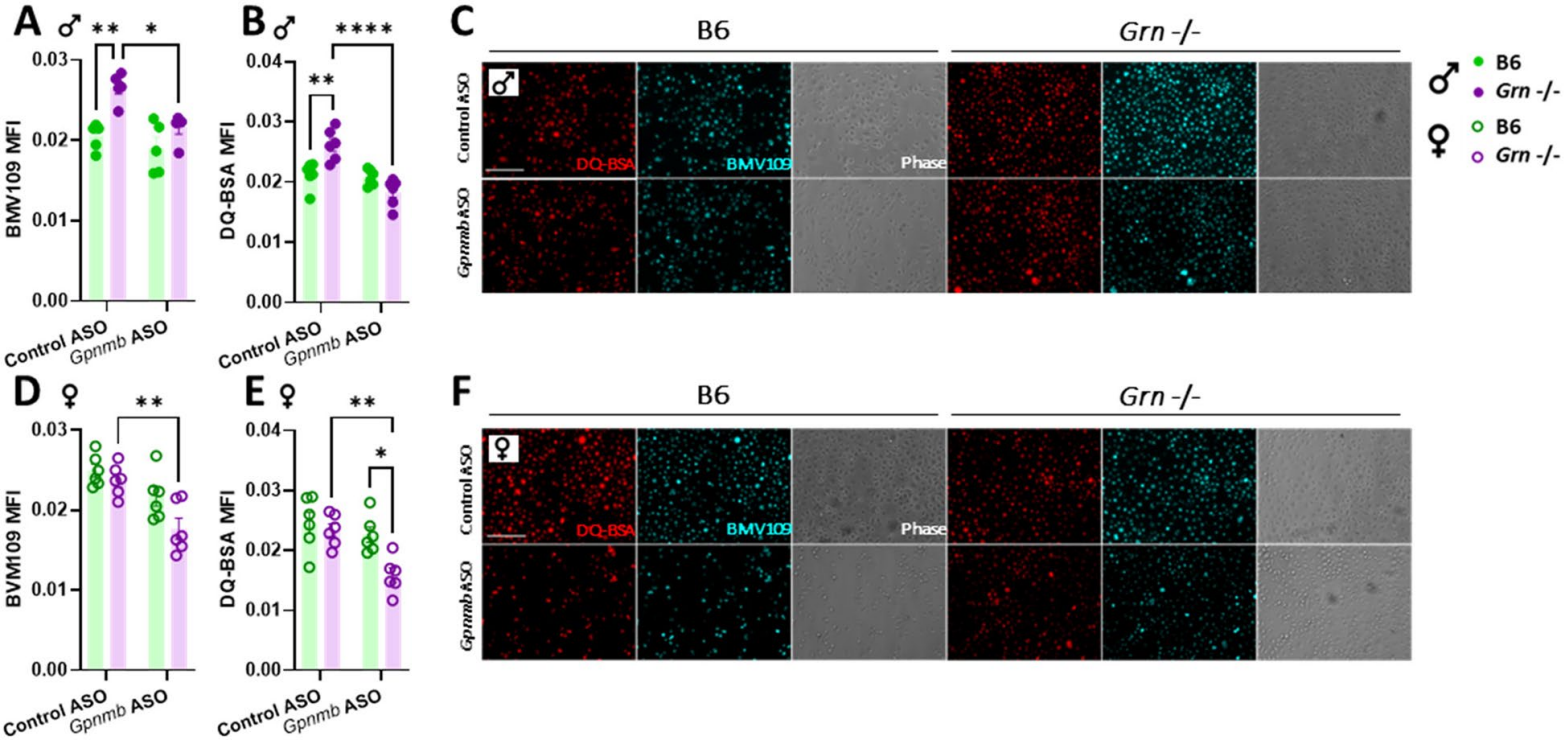
ASO-mediated knock-down of Gpnmb decreases lysosomal protein degradation in *Grn*-deficient macrophages from female mice. pMacs from B6 and *Grn* -/-, 5-to-6-month-old, male and female mice were nucleofected with control or *Gpnmb*-targeting ASO, plated and allowed to rest for 24 h and lysosomal function assessed via microscopy. BMV109 and DQ-BSA MFI was quantified from microscopy images of pMacs from male mice (**A**, **B**, **C**). BMV109 and DQ-BSA MFI was quantified from microscopy images of pMacs from female mice (**D**, **E**, **F**). Bars represent mean ± SEM (N = 6 mice per genotype). Two-way ANOVA, Bonferroni post-hoc, * = *p* < 0.05, ** = *p* < 0.01, *** = *p* < 0.005, **** = *p* < 0.001. Scale bars, 30 μM

In order to determine the extent to which Gpnmb increases in *Grn* -/- macrophages to protect lysosomal function, nucleofected pMacs were incubated with the pan-cathepsin probe, BMV109, and DQ-BSA, a probe used to quantify lysosomal protein degradation, and fluorescence levels were quantified. Interestingly, we observed different sex-dependent effects of Grn-deficiency and subsequent Gpnmb knock down in pMacs from males and females. pMacs from *Grn* -/- males exhibited significantly increased BMV109 and DQ-BSA MFI in control ASO conditions, indicative of increased lysosomal function (Fig. 3A-C). When Gpnmb was knocked down via ASO, these lysosomal readouts were reduced to levels comparable to B6 pMacs. In contrast, pMacs from *Grn* -/- females displayed minimal lysosomal phenotypes at baseline, but upon Gpnmb knock down deficits were revealed as indicated by decreases BMV109 and DQ-BSA relative to B6 controls (Fig. 3D-F). These same sex-specific phenotypes were also observed with lysotracker, a general marker for sufficiently acidic lysosomes, in *Grn -/-* pMacs (Sup. Figure 4A-D). It seems, therefore, that in *Grn* -/- females, Gpnmb increases to protect lysosomal function, and once knocked down, loss of Gpnmb predisposes lysosomes to the dysfunction driven by PGRN deficiency. This is in contrast to macrophages from *Grn* -/- males which, although exhibiting decreased lysosomal function upon Gpnmb knock-down, seem to be protected from lysosomal function due to higher levels of pan cathepsins and lysosomal degradative capacity at baseline, presumably driven by increased Gpnmb levels. Importantly, manipulation of Gpnmb expression via ASO did not affect lysosomal function in pMacs isolated from B6 mice of either sex in the absence of GRN deficiency.

### ASO-mediated knock-down of *Gpnmb* increases stimulation-dependent MHC-II surface expression, but not MHC-II processing in *Grn*-deficient macrophages in a sex-dependent manner

**Fig. 4:**
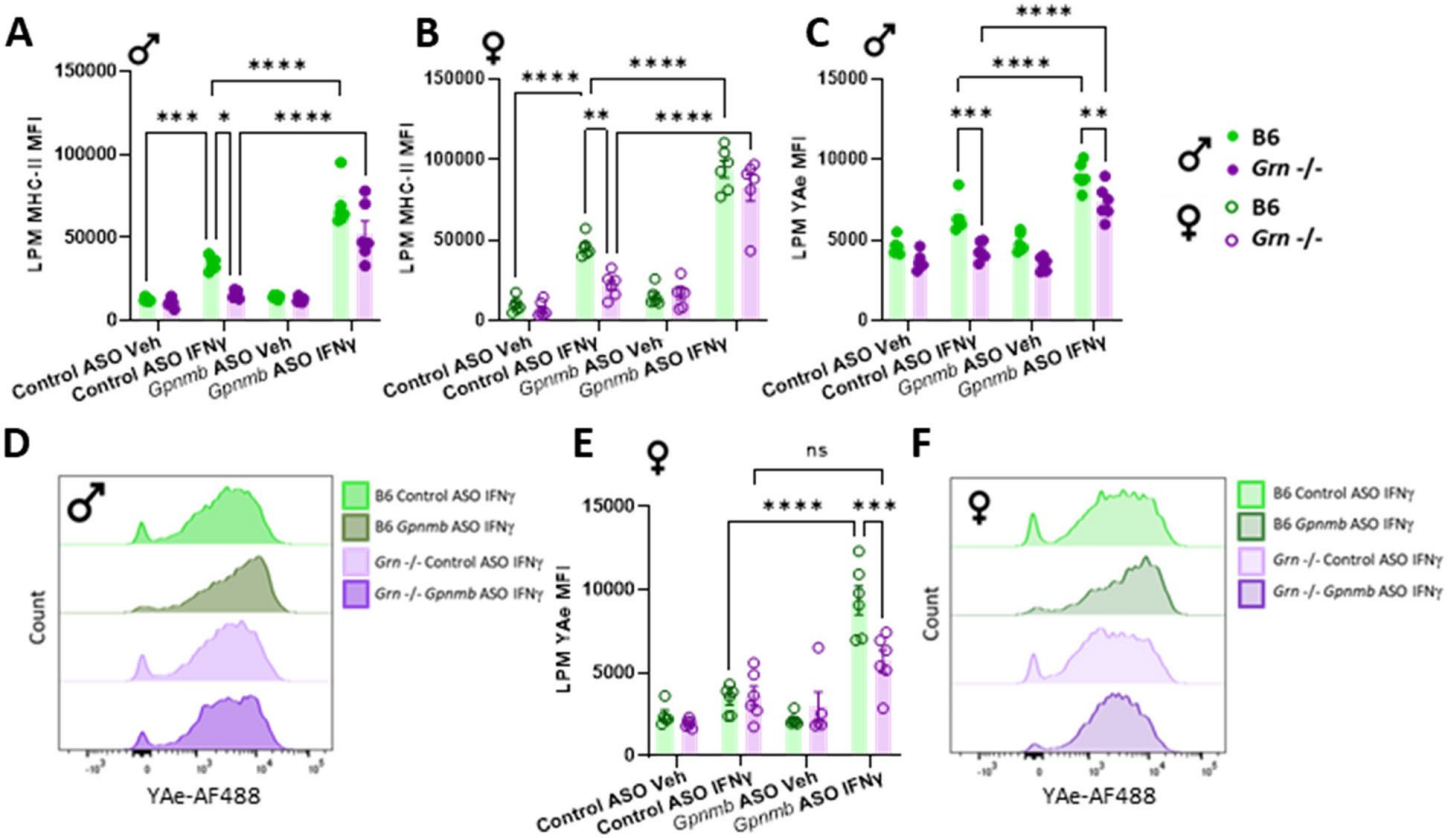
ASO-mediated knock-down of Gpnmb increases MHC-II surface expression but not MHC-II processing in macrophages from *Grn*-deficient females. pMacs from B6 and *Grn* -/-, 5-to-6-month-old, male and female mice were nucleofected with control or *Gpnmb*-targeting ASO, plated and allowed to rest for 24 h, followed by 18-h incubation in presence or absence of 100 U IFNγ and cells assessed via flow cytometry. MHC-II MFI was quantified in MHC-II + LPMs from male and female mice (**A**, **B**). Cells were incubated with Eα peptide and peptide-bound MHC-II complexes at the plasma membrane were quantified in LPMs from male and female mice (**C-F**). Bars represent mean ± SEM (N = 6 mice per genotype). Two-way ANOVA, Bonferroni post-hoc, * = *p* < 0.05, ** = *p* < 0.01, *** = *p* < 0.005, **** = *p* < 0.001

Next, to determine if alterations in lysosomal function were accompanied by alterations in MHC-II surface expression, nucleofected pMacs were treated with 100U IFNγ or vehicle for 18-h, and surface MHC-II expression assessed on LPMs. Similar to what was observed in patient monocytes, in *Grn* -/- LPMs nucleofected with control ASO, a significant decrease in stimulation-dependent MHC-II expression was observed in both sexes relative to B6 controls (Fig. 4A, B). Interestingly, when Gpnmb is knocked down via ASO, an increase in stimulation-dependent MHC-II surface expression was observed in all genotypes and sexes. It seems, therefore, that the increased levels of Gpnmb in *Grn* -/- suppressed stimulation-dependent immune responses, while decreased levels of Gpnmb increased these responses. Indeed, as previously mentioned, Gpnmb ECF acts as a paracrine factor to affect neighboring cells for modulating immunosuppression, and Gpnmb ECF is elevated in media from *Grn* -/- macrophages in both vehicle and IFNγ treatments (Fig. 2G, H); it therefore may be increased Gpnmb ECF that induces this suppressed response in *Grn* -/- macrophages.

Antigen presentation and MHC-II complex assembly is highly dependent on lysosomal function. However, decreased lysosomal function upon Gpnmb knock down is observed in female *Grn* -/- LPMs with concomitant increases in MHC-II surface expression. It is possible that MHC-II expression is increased on the surface of these cells due to altered processing and uptake of the MHC-II complex. The Eα: YAe model is used to monitor the antigen presentation capabilities of cells by incubating cells with an endogenous peptide (Eα 52–68) which is subsequently phagocytosed, transported to the lysosome, loaded onto an MHC-II complex at the lysosome and transported back to the plasma membrane for antigen presentation (Sup. Figure 4E). This Eα peptide-loaded MHC-II can subsequently be detected using flow cytometry using the YAe antibody [37, 38]. This model allows us to measure antigen presentation of a peptide directly and acts as a measure of the whole antigen presentation pathway, from uptake to peptide loading to presentation. It was observed here that YAe MFI, in control ASO nucleofected LPMs from males, was significantly decreased in *Grn* -/- cells relative to B6 controls (Fig. 4C, D). Upon Gpnmb knock-down, however, stimulation-dependent YAe MFI significantly increased in both genotypes, with male *Grn* -/- LPMs exhibiting decreased YAe MFI relative to B6 controls, suggesting that Gpnmb knock-down increases stimulation-dependent activity of the antigen presenting pathway in macrophages from *Grn* -/- and B6 males. Interestingly, no significant differences were observed between genotypes in control ASO nucleofected LPMs from females (Fig. 4E, F). However, when Gpnmb is knocked down, LPMs from female *Grn* -/- mice exhibited decreased stimulation-dependent YAe MFI relative to B6 controls, suggesting dysregulation of the antigen processing pathway in these cells when Gpnmb is reduced acutely.

### ASO-mediated knock-down of *Gpnmb* decreases MHC-II uptake and recycling in peritoneal macrophages from *Grn*-deficient females

**Fig. 5:**
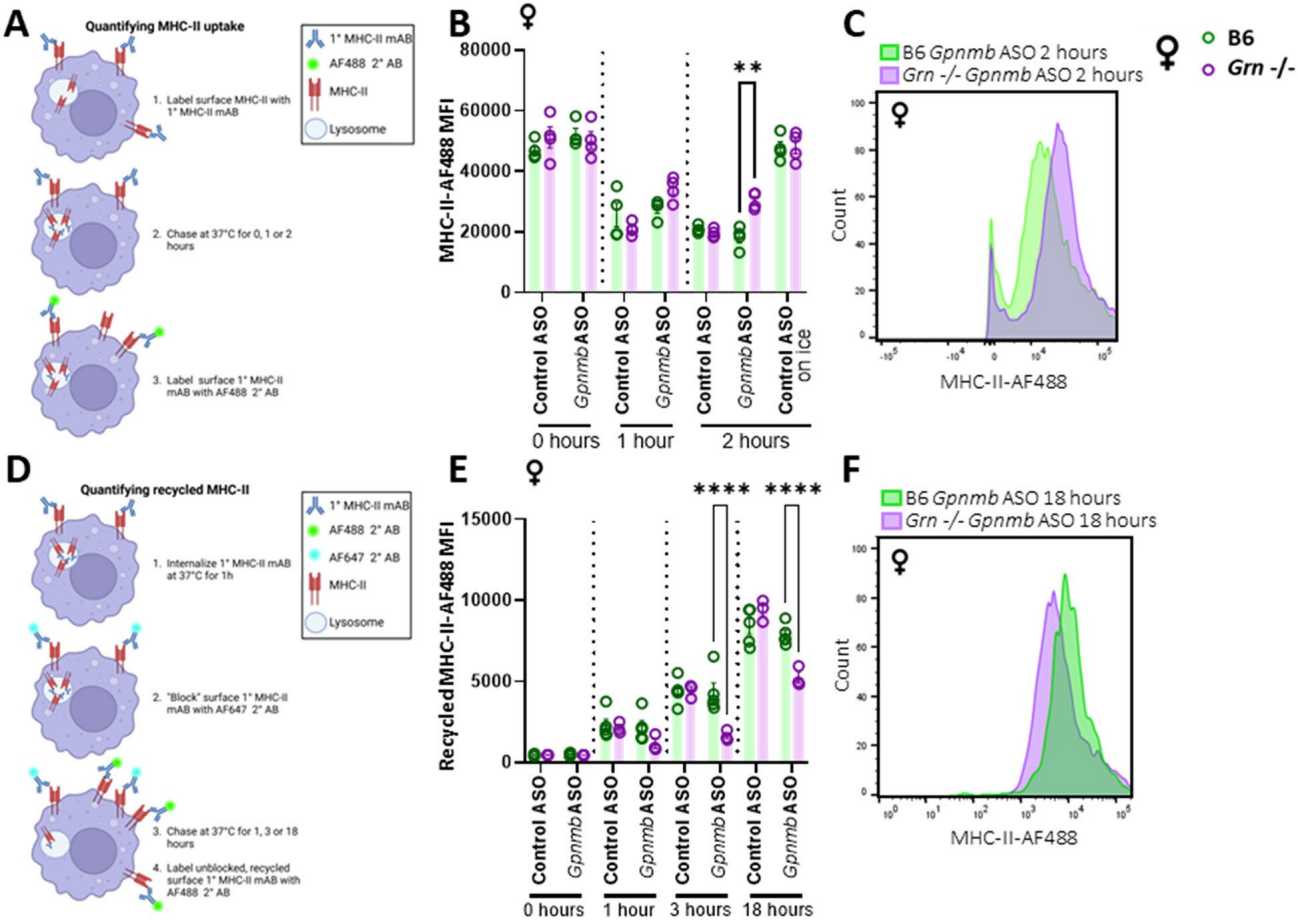
ASO-mediated knock-down of Gpnmb decreases MHC-II uptake and recycling in macrophages from *Grn*-deficient females. pMacs from B6 and *Grn* -/-, 5-to-6-month-old, female mice were nucleofected with control or *Gpnmb*-targeting ASO, plated and allowed to rest for 24 h. After which, they were assessed for MHC-II uptake utilizing a pulse-chase flow cytometry-based assay (**A**). MHC-II-488 MFI was quantified in LPMs from female mice over a 2-h time-course, with an ‘on ice’, no-endocytosis control included (**B**, **C**). pMacs were assessed for MHC-II recycling utilizing a pulse-chase flow cytometry-based assay (**D**). Recycled MHC-II MFI was quantified in LPMs from female mice over an 18-h time-course (**E**, **F**). Bars represent mean ± SEM (N = 6 mice per genotype). Three-way ANOVA, Bonferroni post-hoc, * = *p* < 0.05, ** = *p* < 0.01, *** = *p* < 0.005, **** = *p* < 0.001

Next, to further determine if altered YAe surface expression upon Gpnmb knock down in *Grn* -/– deficient pMacs (Fig. 4) is due to altered MHC-II processing, uptake and recycling of MHC-II complexes were quantified in pMacs in the presence of 100U IFNγ via flow cytometry. MHC-II uptake was quantified via flow cytometry by utilizing a previously described pulse-chase approach [33]. Briefly, surface MHC-II complexes were labelled with a monoclonal primary antibody (pulse) and allowed to be internalized by the macrophage for 0, 1 or 2 h (chase; Fig. 5A). At each timepoint, surface MHC-II remaining was detected via fluorescent secondary antibody. A significant decrease in surface MHC-II was seen overtime as would be expected (Fig. 5B). Control cells left on ice for 2 h (no-endocytosis control) showed no significant decrease in surface MHC-II, indicating no uptake of MHC-II. At both the 0- and 1-h time point, no significant differences were observed between genotypes. However, at the 2-h time point, a significant increase in MHC-II surface expression was observed when Gpnmb was knocked down in *Grn* -/- pMacs from females relative to B6 controls and ASO control groups (Fig. 5B,C). These observations indicate that uptake of MHC-II complex from the plasma membrane was disrupted in the absence of Gpnmb in *Grn* -/- female pMacs.

Next, to determine if recycling of MHC-II back up to the cell surface once internalized is also impaired, we then used a previously published pulse-chase method [39]. As with the uptake assay, surface MHC-II was first labelled with primary monoclonal antibody (Fig. 5D) This labelled MHC-II was then allowed to be internalized for 1 h at 37 °C (pulse). A secondary fluorescent antibody was used to block any remaining labelled MHC-II on the cell surface. Internalized, labelled MHC-II was then ‘chased’ for 1-, 3- or 18-h in the presence of IFNγ. At the end of each time-point, recycled MHC-II that returned to the surface was detectable via fluorescent secondary antibody. A significant increase in recycled MHC-II expression was seen across timepoints as expected (Fig. 5E). At the 0- and 1-h timepoints, no significant differences were observed between genotypes or nucleofection conditions. However, at both the 3- and 18-h time points, a significant reduction in recycled MHC-II was observed in female *Grn* -/- pMacs nucleofected with* Gpnmb*-targeting ASO relative to B6 controls and control ASO conditions (Fig. 5E, F). No significant differences between genotypes were seen in male pMacs, suggesting that, in males, the decrease in Gpnmb does not significantly reduce MHC-II uptake and recycling in PGRN-deficient cells as it does in females (Sup. Figure 4F, G), which is consistent with the Yae data described herein (Fig. 4C-F).

### IL6 secretion increases following ASO-mediated *Gpnmb* knock-down in *Grn*-deficient mouse peritoneal macrophages

**Fig. 6:**
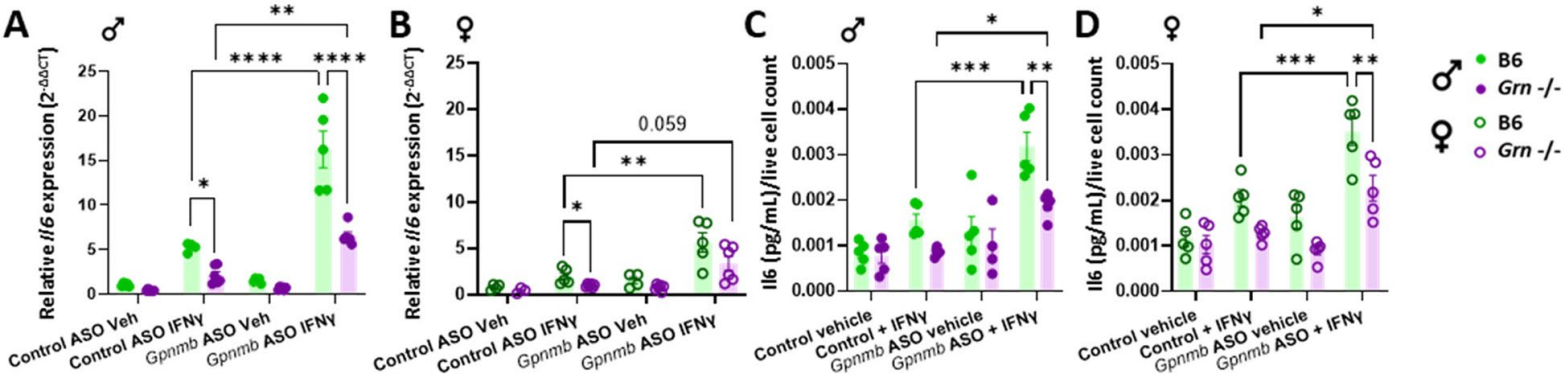
ASO-mediated knock-down of Gpnmb dysregulates stimulation-evoked cytokine transcription and protein secretion. pMacs from B6 and *Grn* -/- female and male mice were nucleofected with control or *Gpnmb*-targeting ASO, plated and allowed to rest for 24 h. After which, they were subject to 18-h incubation in presence or absence of 100 U IFNγ and cell RNA extracted and conditioned media collected. *IL6* transcript levels were assessed in pMacs from male and female mice (**A**, **B**). IL6 cytokine release in media was quantified in conditioned media and normalized to live cell count in pMacs from male and female mice (**C**, **D**). Bars represent mean ± SEM (N = 5–6 mice per genotype). Two-way ANOVA, Bonferroni post-hoc, * = *p* < 0.05, ** = *p* < 0.01, *** = *p* < 0.005, **** = *p* < 0.001

Alterations in Gpnmb expression have been shown to modulate cytokine expression and release in various immune cell subtypes [17]. To determine how ASO-mediated knock-down of Gpnmb would modulate cytokine levels in the context of PGRN deficiency, cytokine mRNA transcripts were quantified as well as cytokines secreted into cell culture media. In both female and male pMacs, a significant reduction in stimulation-dependent transcript levels of *Il6* was observed in *Grn* -/- pMacs in control ASO conditions (Fig. 6A, B). Interestingly, upon knock-down of Gpnmb, a significant increase in stimulation-dependent *Il6* production was observed in both male and female B6 pMacs, and *Grn* -/- male pMacs (Fig. 6A, B). pMacs from with *Grn* -/- female mice exhibited similar phenotypic effects of Gpnmb knock-down, although this did not reach statistical significance (p = 0.059). Interestingly, *Il6* levels were still significantly decreased in male *Grn* -/- pMacs nucleofected with *Gpnmb*-targeting ASO relative to B6 controls. A similar phenotypic pattern was observed regarding Il6 secretion into the cell culture media (Fig. 6C, D). Although a clear effect of genotype was not consistently observed in other cytokines quantified (Sup. Figure 5), a significant increase in cytokine transcript levels and/or production were observed upon Gpnmb knock-down of in both sexes and genotypes, suggesting that Gpnmb may have an immunosuppressive effect on macrophage cytokine production and release.

## Discussion

Our previous work revealed that GPNMB expression is significantly upregulated in peripheral myeloid cells of young PGRN-deficient mice and much earlier than in the CNS [18, 19]. However, the reason for and the effects of these GPNMB increases in these diseases is still unknown and has important implications for approaches aimed at targeting GPNMB as a therapy in neurodegenerative diseases. Here we demonstrate that, in patient monocytes and murine macrophages, GPNMB increases in the context of *GRN* mutations or *Grn* deficiency, and GPNMB knock-down predisposes peripheral patient myeloid cells and *Grn* -/- macrophages from female mice to lysosomal deficits. Furthermore, GPNMB upregulation is associated with a decrease in MHC-II expression. Interestingly, subsequent loss of Gpnmb in both B6 and *Grn* -/- macrophages results in increased MHC-II at the cell surface; however, the mechanism underlying this Gpnmb-dependent increase in MHC-II surface expression appears to be sex- and genotype-dependent. Specifically, in macrophages from both male and female B6 mice as well as male *Grn* -/- mice, MHC-II surface expression increases in the absence of Gpnmb, presumably due to the decrease in Gpnmb ECF, which is known to exert an immunosuppressive effect on immune cells [27, 28]. In contrast, in macrophages from female *Grn* -/- mice, loss of Gpnmb causes decreased MHC-II complex uptake and recycling, presumably due to the lysosomal dysfunction induced by acute removal of Gpnmb, causing MHC-II complexes to accumulate on the cell surface. Lastly, increases in GPNMB leads to a decrease in stimulation-dependent Il1β and IL6 cytokine secretion in *GRN*-FTD PBMCs and *Grn* -/- pMacs, respectively, with GPNMB knock-down increasing expression and secretion of these cytokines. This increase in pro-inflammatory cytokine secretion may be driven by the decrease Gpnmb ECF and its immunosuppressive function.

GPNMB has been hypothesized to increase in the context of neurodegeneration due to lysosomal dysfunction. Indeed, our findings herein suggest that GPNMB increases in both FTD-*GRN* carrier monocytes and *Grn* -/- female mouse macrophages to protect lysosomal function. Specifically, the knock-down of this important regulator of lysosomal function results in robust and significant lysosomal dysfunction in *GRN* mutant and *Grn* -/- cells from female mice. It has previously been demonstrated that *Grn* -/- mice develop lipofuscinosis and lysosomal swelling in an age-dependent manner [40–42]. Interestingly, lysosomal phenotypes have thus far only been reported in the brains of these mice at the earliest time-point of 10 months [40]. It is interesting, therefore, that we observed lysosomal deficits in *Grn* -/- macrophages from 5-to-6-month-old female mice only upon Gpnmb knock-down. As this is also the earliest time-point at which Gpnmb upregulation has been reported in murine preclinical models [19], taken together our data suggest that Gpnmb upregulation may be a compensatory mechanism to rescue and protect the lysosome in the absence of PGRN, and that this occurs much earlier in the peripheral immune system than in the CNS. Although it was demonstrated that altered lysosomal acidity (a reliable indicator of lysosomal health) was observed upon GPNMB knock-down in *GRN*-FTD patient monocytes, it was not possible to confirm lysosomal dysfunction with an orthogonal assay (such as BMV109 or DQ-BSA) as was done here in murine macrophages due to a limited supply of cryopreserved cells available. Future studies will be needed to confirm lysosomal dysfunction in *GRN*-FTD patient monocytes upon GPNMB knock-down.

Antigen presenting cells, such as macrophages, are highly dependent on lysosomal function for efficient peptide loading and subsequent antigen presentation [43–45]. The decrease in lysosomal degradation and cathepsin activity in female *Grn* -/- macrophages and increase in lysosomal acidity in *GRN-*FTD PBMCs observed herein upon Gpnmb knock-down supports a model in which the reported Gpnmb upregulation in PGRN-deficient mice [18–20] is a compensatory response to protect the lysosome in the absence of PGRN. In female *Grn* -/- macrophages, knock-down of Gpnmb also mediated MHC-II complex cell surface expression and the ability of these *Grn* -/- macrophages to engage in antigen presentation. From our findings it is difficult to discern whether the accumulation of MHC-II at the cell surface upon Gpnmb knock-down in *Grn* -/- macrophages from female mice represent newly synthesized MHC-II complexes or peptide-bound MHC-II; however, given that a decrease in Eα peptide-bound MHC-II expression was observed in *Grn* -/- macrophages from female mice upon Gpnmb knock-down, the former possibility is more likely, but further research is required to conclusively distinguish between these two possibilities.

We demonstrated here that, although Gpnmb protein and RNA levels are upregulated in pMacs from both male and female *Grn* -/- mice, knock-down of Gpnmb only reveals lysosomal deficits and dysregulated MHC-II processing in pMacs from females. Therefore, it seems that male *Grn* -/- mice have additional compensatory mechanisms protecting lysosomal function which females do not possess. Interestingly, sex-specific differences have been reported in patients with neurodegenerative diseases regarding lysosomal function and gene expression [46, 47]. Sex differences in the regulation of the autophagosome–lysosome system in particular have been proposed to modulate the severity of neurodegenerative disorders, because prior studies suggest that women have lower basal autophagy levels [48]. Such reports may explain why macrophages from female *Grn* -/- mice were vulnerable to lysosomal dysfunction when Gpnmb was knocked down. Unlike Alzheimer’s Disease, which is overrepresented in females [49], FTD prevalence is found to be similar in men and women [50]. However, sex has been found to influence clinical phenotype in FTD, with the behavioral variant of FTD reported to be more common in men, whereas primary progressive aphasia is overrepresented in women [51]. The distribution of clinical phenotypes in the human cohort assessed in this report were relatively evenly distributed between males and females (Table 9). Furthermore, this cohort was underpowered to stratify participant data by sex, therefore we are unable to comment on the effect of sex and clinical phenotypes on the cellular phenotypes reported here. Whether differences in vulnerability of the lysosomal system underly the reported sex-differences in clinical phenotypes in FTD is of interest for future research.

**Table 9:**
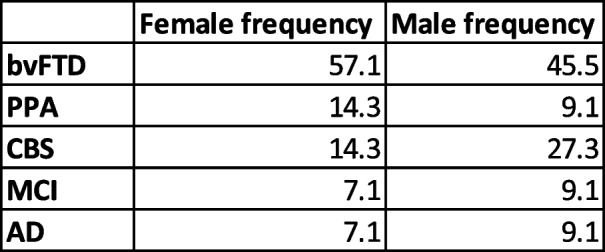
Distribution of clinical phenotypes between male and female patients. bvFTD = Behavioral variant frontotemporal dementia, PPA = Primary progressive aphasia, CBS = Corticobasal syndrome, MCI = Mild Cognitive Impairment, AD = Alzheimer’s Disease

Previous studies have reported heightened immune responses and inflammation in Grn-deficient preclinical models [18, 30], which contrast with the decreased cytokine release and MHC-II expression observed in this study. Several reasons may underlie these conflicting observations in the literature. In the 2010 pioneering paper, in vivo data was collected from 18-month-old mice, significantly older than the 5-to-6-month-old used here. Furthermore, the ex vivo macrophage data reported by Yin et al. was collected from bone-marrow-derived macrophages (BMDMs) from 2-month-old mice. When using BMDMs, bone marrow is isolated from mouse femurs and stem cells are differentiated to macrophages ex vivo, which contrasts with the pMacs used here which are differentiated in vivo. pMACs therefore provide a readout of the responsiveness of the innate immune system in the background of the animal from which they are isolated, particularly informative for knockout or genetically modified animals, and is even more imperative when assessing effects of genotype at different ages which cannot be captured by BMDMs that are differentiated ex vivo, outside of the context of the ageing animal [52]. We have observed that the upregulation of GPNMB in *Grn* -/- mice does not occur in the periphery until 5-to-6 months of age and cannot be observed in 2-to-3-month-old mice (Sup. Figure 6). It is likely, therefore, that studies utilizing macrophages will not detect the immunosuppressive effects of Gpnmb in young *Grn*-deficient mice as Gpnmb becomes upregulated at later ages.

GPNMB has been discussed as a potential therapeutic target in PGRN-mediated neurodegeneration [20]. Specifically, GPNMB was proposed as a potential therapeutic target for interrupting the transmission of pathological α-synuclein in PD based on observations that GPNMB concentration in plasma positively correlated with UPDRS score, and knock-down of *GPNMB* in iPSC-derived neurons reduced α-synuclein pre-formed fibril (PFF) uptake [53]. However, based on our novel findings herein we would argue that the reported positive correlation between GPNMB expression and UPDRS score is likely driven by increased lysosomal dysfunction as disease progresses, with GPNMB increasing to restore lysosomal function. Given that Gpnmb ECF has previously been reported to suppress inflammatory activity [17], it is likely that the Gpnmb ECF increases in *Grn* -/- deficient macrophages reported here occur to curb the cytokine and MHC-II responses, however further research is required to understand the exact mechanisms in which Gpnmb ECF exerts these effects. Based on reports that GPNMB can exert an anti-inflammatory effect by promoting inflammation resolution and our observations that increased GPNMB expression is associated with reduced cytokine release and MHC-II expression with knock-down ameliorating both, we propose an alternative strategy: that lysosomal function in FTD and other neurodegenerative diseases where it is impaired may be rectified by GPNMB therapy. Yet, a cautionary note to be considered in future studies is that an excessively dampened immune response may be as deleterious as an overactive one and could accelerate neurodegeneration [54].

## Conclusions

Our findings reported herein are consistent with a model in which GPNMB is upregulated in the absence of PGRN to protect lysosomal function, and with this comes a dampening of the immune system possibly to cap or resolve inflammatory challenges. Considering FTD-*GRN* patients carry a single copy of an FTD-associated mutation, one limitation of the current study is the use of *Grn* -/- mice to study the interplay between Pgrn and Gpnmb. Moving forward, it will be important to determine if the phenotypes observed in *Grn* -/- mice are also observed in the arguably more disease-relevant *R493X* knock-in pre-clinical model of FTD [55].

The sex-dependent differences observed in preclinical animal models of disease were not confirmed in patient samples in this study, a limitation of this study that should be addressed in future research. However, this sexual dimorphism suggests that future therapeutic development should consider relative sex differences when aiming for personalized precision medicine. Moreover, although upregulation of GPNMB in young (5-to-6-month-old) Grn-deficient mice is associated with improved lysosomal function, we still do not know how age modulates these phenotypes, or how the protective role that upregulation of GPNMB may afford in the context of FTD-*GRN* mutant carriers is modified by an ageing immune system. One important implication of the findings herein is that caution should be taken when targeting GPNMB for potential therapeutics, as excessive reduction in GPNMB levels or activity may put lysosomal function at risk and increase the pro-inflammatory milieu in the central-peripheral neuroimmune crosstalk which would compromise brain health [56]. Future studies will be needed to determine the extent to which strategies to increase GPNMB levels using small molecules or gene therapy may serve to rescue or protect the lysosome from further dysfunction and cap or curtail detrimental chronic inflammatory responses centrally and peripherally.

## Supplementary Information

Supplementary Material 1.

Supplementary Material 2.

## Data Availability

The datasets generated and analyzed during the current study are available in the Zenodo repository, 10.5281/zenodo.12773066.

## Abbreviations

PGRN: Progranulin
FTD: Frontotemporal dementia
LSD: Lysosomal storage disorder
AD: Alzheimer’s Disease
PD: Parkinson’s Disease
CNS: Central nervous system
GPNMB: Glycoprotein non-metastatic B
ASO: Antisense oligonucleotide
NCRAD: National Centralized Repository for Alzheimer’s Disease and Related Dementias
PBMCs: Peripheral blood mononuclear cells
NHCs: Neurologically healthy controls
pMHC-II: Peptide-loaded MHC-II complexes
