# Supplementary material for: ASO-mediated knock-down of GPNMB in mutant-GRN and in Grn-deficient peripheral myeloid cells disrupts lysosomal function and immune responses
Source: Mol Neurodegener. 2025 Apr 8;20:41. doi: 10.1186/s13024-025-00829-w (PMC11980231; doi:10.1186/s13024-025-00829-w)
Supplement: Supplementary file 1 — Supplementary Material 1. [file 13024_2025_829_MOESM1_ESM.docx]

**Supplementary Material**

**Supplementary figures**

**
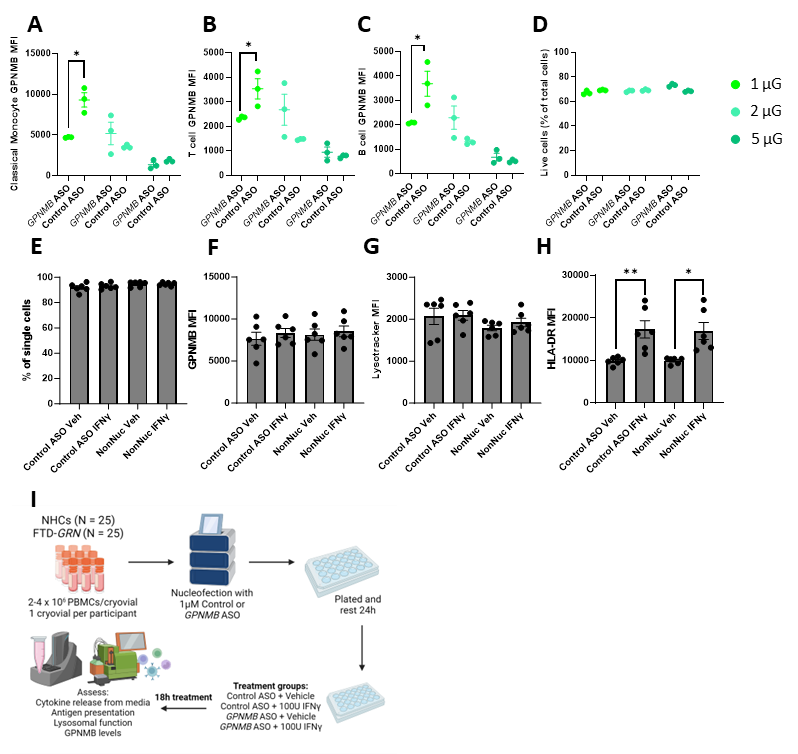
**

**Supplementary Figure 1. Optimization of GPNMB knock-down in human PBMCs.** PBMCs from NHCs were nucleofected with 1, 2 or 5μg *GPNMB*-targeting or control ASO and assessed for GPNMB MFI via flow cytometry (**A, B, C, D**). PBMCs from NHCs were nucleofected with 1μg control ASO or left non-nucleofected and assessed for live cell frequency (**E**), GPNMB MFI (**F**), Lysotracker MFI (**G**) and HLA-DR MFI (**H**) via flow cytometry. Bars represent mean +/- SEM (N = 3-6 participants per treatment group) One/Two-way ANOVA, Bonferroni post-hoc, * = p < 0.05, ** = p < 0.01, *** = p < 0.005, ****= p < 0.001. Schematic of experimental design: PBMCs from NHCs and FTD-GRN patients were nucleofected with control or GPNMB-targeting ASO, plated and allowed to rest for 24 hours, followed by 18-hour incubation in presence or absence of 100 U IFNγ and cells assessed via flow cytometry and media taken for cytokine quantification (**I**).

**
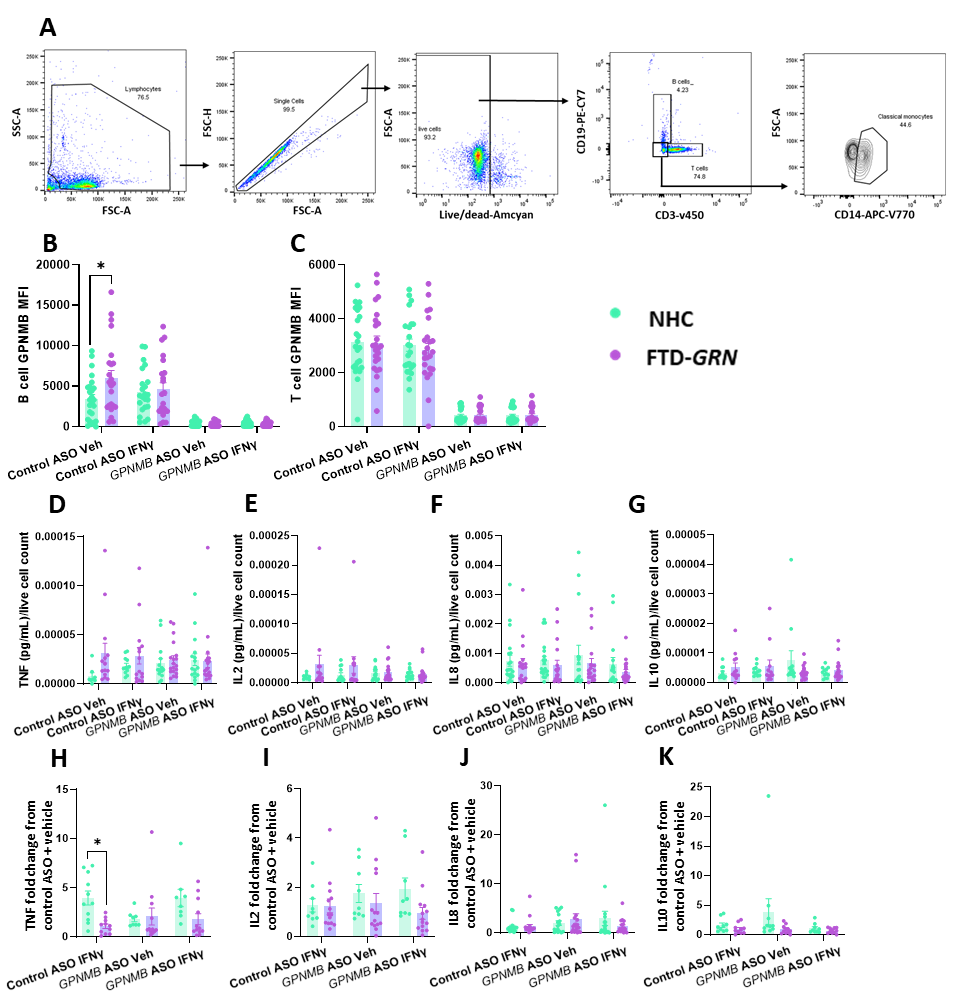
**

**Supplementary Figure 2. Immunophenotyping PBMCs and cytokine release.** PBMCs from NHCs and FTD-*GRN* patients were nucleofected with control or *GPNMB*-targeting ASO, plated and allowed to rest for 24 hours, followed by 18-hour incubation in presence or absence of 100U IFNγ and cells assessed via flow cytometry and media taken for cytokine quantification. (**A**) Schematic of flow cytometry gating strategy. GPNMB MFI was quantified in B and T cells (**B, C**). Cytokine release was quantified in media and normalized to live cell count (**D-G**). Cytokine release was quantified in media, normalized to live cell count and fold-change from control ASO vehicle conditions calculated (**H-K**). Bars represent mean +/- SEM (N = 20-25 participants per disease state) Two-way ANOVA, Bonferroni post-hoc, * = p < 0.05, ** = p < 0.01, *** = p < 0.005, ****= p < 0.001.

**
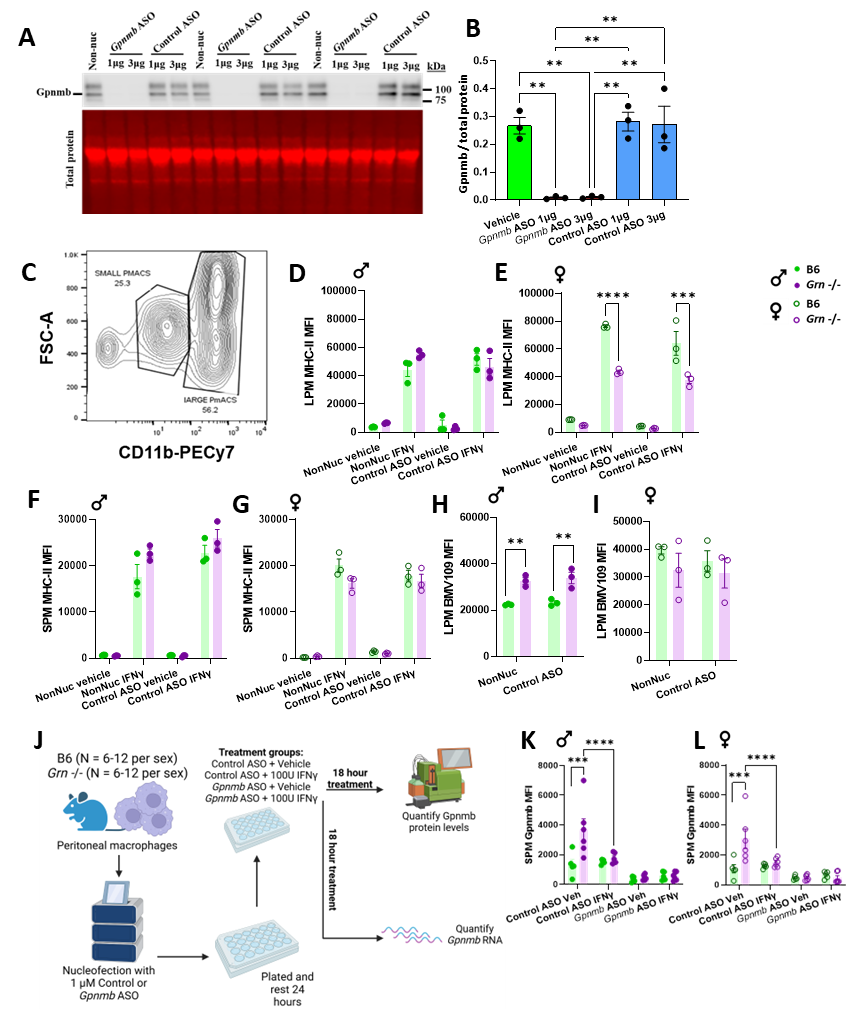
**

**Supplementary Figure 3. Optimization of Gpnmb knock-down in murine pMacs.** pMacs from 5-to-6-month-old B6 mice were nucleofected with 1 or 3μg control or *Gpnmb*-targeting ASO or left non-nucleofected and Gpnmb protein expression assessed via western blot (**A, B**). Schematic of pMac gating strategy to distinguish LPM and SPM based on Cd11b expression (**C**). pMacs from B6 and *Grn* -/-, 5-to-6-month-old, male and female mice were nucleofected with 1μg control ASO or left non-nucleofected and assessed for MHC-II MFI and lysosomal function via flow cytometry (**D-I**). Schematic of experimental design: pMacs from B6 and Grn -/-, male and female mice were nucleofected with control or Gpnmb-targeting ASO, plated and allowed to rest for 24 hours, followed by 18-hour incubation in presence or absence of 100 U IFNγ and cells assessed via flow cytometry or RNA extracted (**J**). pMacs from B6 and Grn -/-, 5-to-6-month-old, male and female mice were nucleofected with control or *Gpnmb*-targeting ASO, plated and allowed to rest for 24 hours. After which, they were assessed for Gpnmb MFI on SPMs. Bars represent mean +/- SEM (N = 3-6 mice per genotype/treatment) One/Two-way ANOVA, Bonferroni post-hoc, * = p < 0.05, ** = p < 0.01, *** = p < 0.005, ****= p < 0.001.


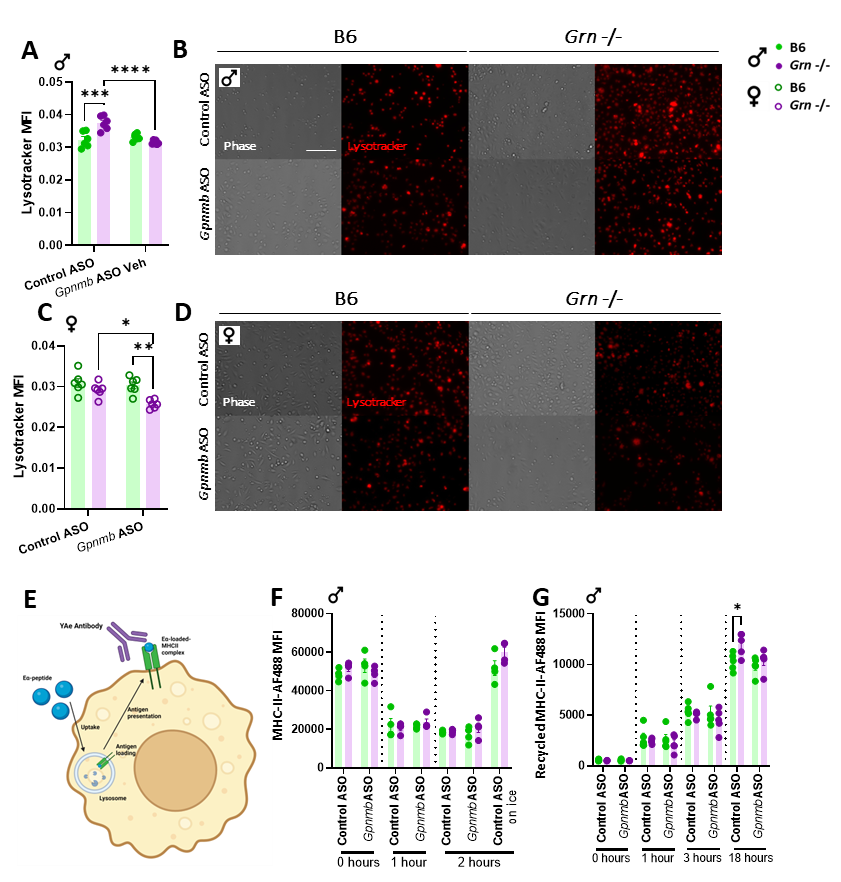


**Supplementary Figure 4. Lysotracker microscopy and MHC-II processing in pMacs.** pMacs from B6 and *Grn* -/-, 5-to-6-month-old, male and female mice were nucleofected with control or *Gpnmb*-targeting ASO, plated and allowed to rest for 24 hours and lysosomal function assessed via microscopy. Lysotracker MFI was quantified from microscopy images of pMacs (**A - D**). Scale bars, 30 μM. (**E**) Schematic of YAe flow-cytometry based assay**.** pMacs from B6 and *Grn* -/-, 5-to-6-month-old, male mice were nucleofected with control or *Gpnmb*-targeting ASO, plated and allowed to rest for 24 hours. After which, they were assessed for MHC-II uptake utilizing a pulse-chase flow cytometry-based assay. MHC-II-488 MFI was quantified in LPMs from male mice over a 2-hour time-course, with an ‘on ice’, no-endocytosis control included (**F**). pMacs were assessed for MHC-II recycling utilizing a pulse-chase flow cytometry-based assay. Recycled MHC-II MFI was quantified in LPMs from male mice over an 18-hour time-course (**G**). Bars represent mean +/- SEM (N = 4 - 6 mice per genotype). Three-way ANOVA, Bonferroni post-hoc, * = p < 0.05, ** = p < 0.01, *** = p < 0.005, ****= p < 0.001.


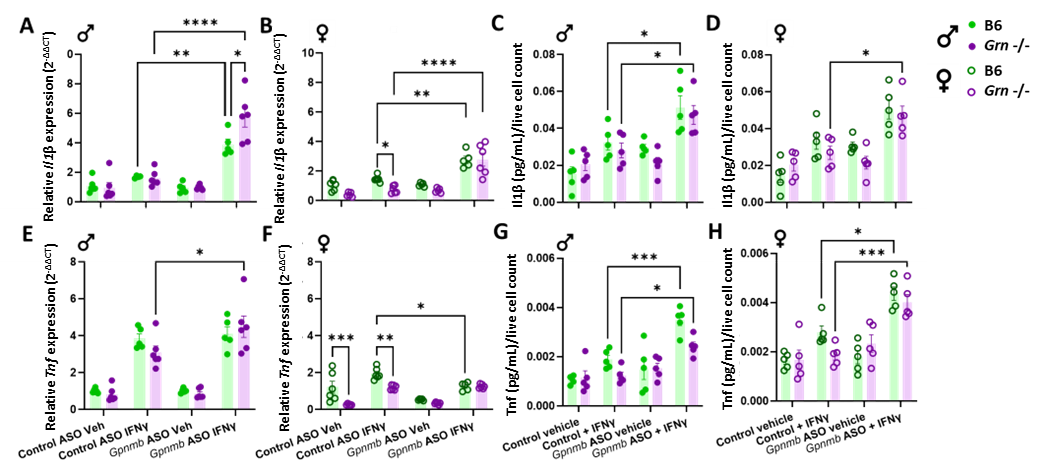


**Supplementary Figure 5. Gpnmb regulates cytokine transcription and secretion in macrophages.** pMacs from B6 and *Grn* -/- female and male mice were nucleofected with control or *Gpnmb*-targeting ASO, plated and allowed to rest for 24 hours. After which, they were subject to 18-hour incubation in presence or absence of 100U IFNγ and cell RNA extracted and media collected. *Il1β* trasncript levels were assessed in pMacs from male and female mice (**A, B**). IL1β cytokine release in media was quantified in media and normalized to live cell count in pMacs from male and female mice (**C, D**). *Tnf* trasncript levels were assessed in pMacs from male and female mice (**E, F**). Tnf cytokine release in media was quantified in media and normalized to live cell count in pMacs from male and female mice (**G, H**). Bars represent mean +/- SEM (N = 5-6 mice per genotype). Two-way ANOVA, Bonferroni post-hoc, * = p < 0.05, ** = p < 0.01, *** = p < 0.005, ****= p < 0.001.

**
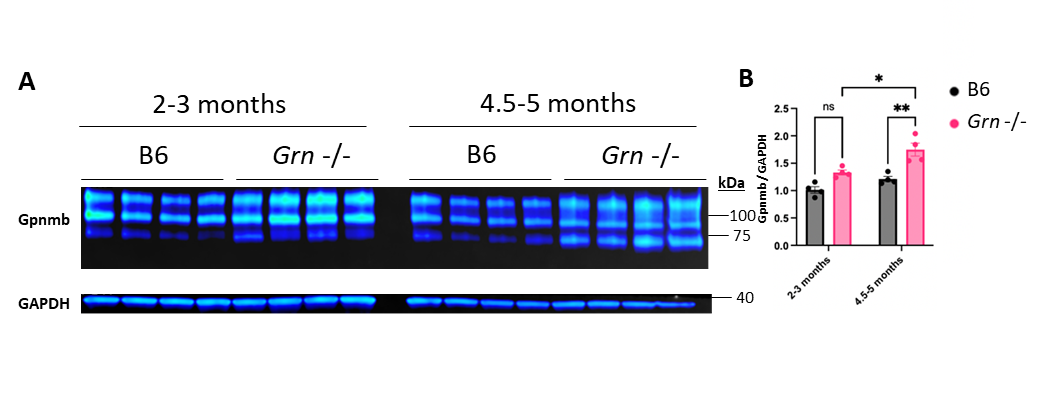
Supplementary Figure 6. Upregulation of Gpnmb can only be observed in *Grn* -/- pMacs once mice age to 4.5-months.** pMacs from B6 and *Grn* -/- female and male mice, aged 2-3 or 4.5-5 months, were lysed and Gpnmb expression quantified and normalized to GAPDH via western blotting (**A, B**). Bars represent mean +/- SEM (N = 4 mice per genotype and age). Two-way ANOVA, Bonferroni post-hoc, * = p < 0.05, ** = p < 0.01.
